# New neotropical species of Opiinae (Hymenoptera, Braconidae) reared from fruit-infesting and leaf-mining Tephritidae (Diptera) with comments on the *Diachasmimorpha mexicana* species group and the genera *Lorenzopius* and *Tubiformopius*

**DOI:** 10.3897/zookeys.243.3990

**Published:** 2012-11-16

**Authors:** Robert Wharton, Lauren Ward, Istvan Miko

**Affiliations:** 1 Department of Entomology, Texas A&M University, College Station, Texas 77843 U.S.A.; 2 Department of Entomology, Pennsylvania State University, 501 ASI Building, University Park, Pennsylvania 16802 U.S.A.

**Keywords:** Parasitoid, classification, *Rhagoletis*, HAO, *Opius*

## Abstract

Four new species of opiine Braconidae are described from Mexico. These are *Diachasmimorpha martinalujai* Wharton reared from *Rhagoletis* infesting fruits of *Crataegus* spp., *Diachasmimorpha norrbomi* Wharton reared from *Euphranta mexicana* infesting fruits of *Ribes pringlei*, *Eurytenes (Stigmatopoea) norrbomi* Wharton reared from *Trypeta concolor* mining leaves of *Barkleyanthus salicifolia* and *Eurytenes (Stigmatopoea) maya* Wharton reared from *Rhagoletis pomonella* infesting apples and fruits of *Crataegus* spp. Morphological features of the first metasomal segment and occipital carina, useful for placement of these species, are discussed relative to the genera *Diachasmimorpha*, *Eurytenes*, *Lorenzopius*, *Tubiformopius*, and *Opius* s.l. Descriptions and diagnoses are referenced to the Hymenoptera Anatomy Ontology. The following represent new combinations: *Diachasmimorpha hildagensis*, *Lorenzopius euryteniformis*, and *Tubiformopius tubibasis*. Revised diagnoses are provided for *Diachasmimorpha hildagensis*, *Diachasmimorpha mexicana*, *Diachasmimorpha sanguinea*, *Eurytenes* (*Stigmatopoea*), *Lorenzopius*, *Lorenzopius euryteniformis*, *Tubiformopius*, *Tubiformopius tubigaster*, *Tubiformopius tubibasis*, *Opius incoligma*, and *Opius rugicoxis*. Two species groups are delineated within *Lorenzopius* and a key to species of *Diachasmimorpha* occurring in the New World is provided.

## Introduction

The subfamily Opiinae is a diverse assemblage of relatively small braconids that develop as koinobiont endoparasitoids of various cyclorrhaphous Diptera, emerging from the puparium of their hosts. Opiines have long been recognized as a distinct taxon within the Braconidae (Wharton and van Achterberg 2000), but specific features suitable for characterizing them as monophyletic relative to the Alysiinae have proven elusive (Wharton 1988, Quicke and van Achterberg 1990, Wharton et al. 2006). Koinobiont endoparasitism of cyclorrhaphous Diptera, with emergence from the puparium of the host, defines Opiinae+Alysiinae. Alysiines are readily characterized by the presence of exodont mandibles (non-overlapping, with teeth pointing outwardly) and an associated median sulcus on the back of the head (Wharton et al. 2006). Exothecines routinely appear as the sister group to Opiinae+Alysiinae (e.g. Wharton et al. 2006) and the labrum is flattened in Opiinae relative to exothecines (and other cyclostomes). The labrum is reduced in Alysiinae relative to Opiinae and cyclostomes in general. Molecular analyses published to date have provided evidence of monophyly for both Opiinae and Alysiinae when only 2–5 taxa are included in each (Dowton et al. 1998, Belshaw et al. 2000, Dowton et al. 2002) but have yet to resolve the problem completely when significantly more taxa are included (Gimeno et al. 1997; Wharton et al. 2006). There are over 1800 valid species in the Opiinae (Yu et al. 2005) and 116 genus group names (84 of these currently treated as valid by one or more authors) have been applied to various combinations of these species.

Fischer (1972, 1977, 1987) monographed the Opiinae on a world basis. This made the group more accessible for study, and this in turn led to numerous changes in the classification. Fischer (1972) initially recognized 23 genera (excluding the Gnamptodontinae, widely accepted subsequently as a separate subfamily). The number of genera currently accepted as valid varies from 17 (Wharton 1997) to about 24 (Fischer 1987, 1999) to 31 (van Achterberg and Maeto 1990, van Achterberg and Salvo 1997, van Achterberg 2004a, b, 2005). The primary purpose of the present study is to describe new species reared from fruit-infesting and leaf-mining Tephritidae from Mexico in order to broaden our understanding of host relationships within Opiinae. The search for the most appropriate genus group name for two of these species and the discovery of previously misplaced species revealed the need for re-characterization of certain genus-group taxa, and this is a secondary goal of the study.

## Materials and methods

**Specimens**. Reared material of several species, including the four newly described below, was kindly sent for study to the senior author by Martin Aluja and Juan Rull (Instituto de Ecologia, Xalapa, Mexico), Robert Jones (Universidad de Autónoma de Querétaro, Querétaro, Mexico), and Allen Norrbom (USDA Systematic Laboratory, Washington, D. C.). Other specimens used in this study, including type material of previously described species, were borrowed from or examined at the following institutions: American Entomological Institute, Gainesville, Florida, USA (AEIC), Canadian National Collection, Ottawa, Ontario, Canada (CNC), Hungarian Natural History Museum, Budapest, Hungary (HNHM), National Museum of Natural History, Leiden, The Netherlands (RMNH), Naturhistorisches Museum Wien, Vienna, Austria (NHNW), Texas A&M University Insect Collection, College Station, Texas, USA (TAMU), The Natural History Museum, London, England (BMNH), and U. S. National Museum of Natural History, Washington, D. C., USA (USNM).

In the material examined section under each species description, we record label data for the holotype exactly as they appear on the labels. We use a more standardized format for paratypes, additional specimens examined, and published data for other specimens.

**Figures.** Images were acquired digitally using Syncroscopy’s AutoMontage® software, in combination with a ProgRes 3008 digital camera mounted on a Leica MZ APO dissecting microscope. All images were further processed using various minor adjustment levels in Adobe Photoshop® such as image cropping and rotation, adjustment of contrast and brightness levels, color saturation, and background enhancement. Automontage images are available in color and high resolution at http://peet.tamu.edu/projects/8/public/site/wharton_lab/home.

**Database management, digital dissemination, and ontology reference.** Illustrations and free-text diagnoses for morphospecies were assembled in mx, a web-based content management system that facilitates data management and dissemination for taxonomic and phylogenetic works (e.g. Yoder et al. 2006). The mx project is open source, with code and further documentation available at http://sourceforge.net/projects/mx-database/. Data pertinent to this work, including specimen-level data, images, diagnoses, and descriptions, are available at http://peet.tamu.edu/projects/8/public/site/wharton_lab/home.

Morphological terms used in this revision were matched to the Hymenoptera Anatomy Ontology (HAO, Yoder et al. 2010) (Appendix). Identifiers (URIs) in the format http://purl.obolibrary.org/obo/HAO_XXXXXXX represent anatomical concepts in HAO version http://purl.obolibrary.org/obo/hao/2011-05-18/hao.owl. They are provided to enable readers to confirm their understanding of the anatomical structures being referenced. To find out more about a given structure, including images, references, and other metadata, use the identifier as a web-link, or use the HAO:XXXXXXX (note colon replaces underscore) as a search term at http://glossary.hymao.org. For published examples see Wharton et al. (2010) and especially Talamas et al. (2011).

**Terminology and measurements.** Terminology as linked through the HAO (Appendix) largely follows Sharkey and Wharton (1997), with a few additions from Walker and Wharton (2011). For the first metasomal segment (sometimes referred to as the petiole), T1 is the median tergite and S1 is the sternite: the well-sclerotized basal portion of the sternum. S1 is often greatly reduced in opiines but well developed in several of the species treated here. A tendon originates in the propodeum and inserts at the base of T1 medially. The point of insertion, which we have called the dorsal tendon attachment, serves as a convenient point of reference for orientation. The propodeum medially has at least a partial areola in most of the species treated here. Morphologically, this areola may not be strictly homologous with the areola as defined, for example, by Townes (1969) for Ichneumonidae or Sharkey and Wharton (1997, Fig. 8) for Braconidae since in these opiines there is no distinct petiolar area posteriorly. On the mesoscutum, some of the species treated here have a mesoscutal humeral sulcus extending along the lateral margin from the base of the notaulus. When present, it is usually carinately margined laterally, and we have referred to this as the supra-marginal carina in the text below. Wing cells are indicated in Fig. 36; abbreviations for wing veins are indicated in Fig. 16, both following Sharkey and Wharton (1997).

Quantitative data in descriptions are based on 5 individuals of each sex, when available. Measurements largely follow Walker and Wharton (2011). Mesosomal width is the distance across the mesoscutum between the tegula. Width of clypeus was measured at the lateral margin rather than at the anterior tentorial pit. The eye/temple ratio is an important species-level characteristic, but is notoriously difficult to measure consistently because slight repositioning may result in significantly different ratios across this curved surface. The measurements are therefore provided to illustrate relative difference among species, and less emphasis should be placed on the absolute values. In the descriptions below, we have indicated whether eye/temple ratios were calculated from measurements made in dorsal view, lateral view, or both.

## Results and discussion

### Generic placement

The new species described below are placed in the genera *Diachasmimorpha* Viereck and *Eurytenes* Foerster. The basis for these placements, with particular reference to the nature of the occipital carina, characteristics of the first metasomal segment, and tephritid parasitism, are discussed in this section. Diagnoses of relevant taxa and descriptions of the new species follow in the next section, alphabetically by genus.

**Figures 1–4.**
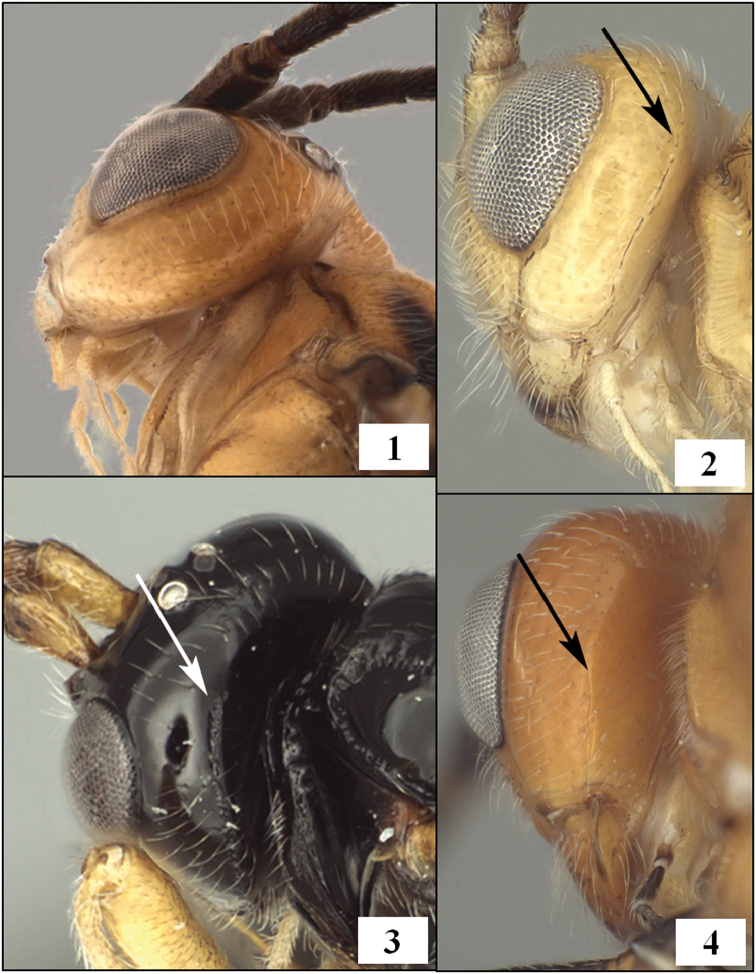
Occipital carina. **1** *Opius (Bellopius) bellus* Gahan, carina completely absent **2** *Diachasmimorpha mellea* (Gahan), arrow at dorsal end of carina **3** *Lorenzopius tubulatus* (Fischer), holotype female, arrow at dorsal end of carina **4** *Diachasmimorpha sanguinea* (Ashmead), arrow at dorsal end of weak carina.

The occipital carina varies from completely present to completely absent in the Opiinae with most species having the carina broadly absent mid-dorsally but well-developed laterally (Figs 1–4). Fischer (1972) created the tribe Desmiostomatini for all species known to him in which the occipital carina was completely lost or apparently so (Fig. 1). Wharton (1983, 1987a, 1988) subsequently discovered that loss of the carina occurred in several other groups as well and hypothesized multiple independent losses within the subfamily. The opiine parasitoids of fruit-infesting Tephritidae are distributed among several genera (Wharton 1997), most of which have at least some species lacking an occipital carina. The New World endemics *Doryctobracon* Enderlein and *Bellopius* Wharton (the latter presently placed as a subgenus of *Opius* Wesmael s.l.) are thus far known only from tephritid hosts and all species lack the occipital carina. The Old World endemics *Psyttalia* Walker and *Fopius* Wharton are also known only as parasitoids of Tephritidae but only a small percentage of the known species have the occipital carina completely lacking. The cosmopolitan *Utetes* Foerster, which may also be restricted to tephritid hosts, contains a group of New World endemics in which the occipital carina is completely lacking. This New World tropical and subtropical group was formerly treated as *Bracanastrepha* Brèthes (Fischer 1977, Wharton 1988). When *Bracanastrepha* was synonymized under *Utetes* (Wharton 1988), and *Utetes* restricted to species with a distinctive hind tibial carina, all of the remaining *Bracanastrepha* that lacked an occipital carina, including those species previously placed in the subgenera *Thiemanastrepha* Fischer and *Buckanastrepha* Fischer, were transferred to *Opius* s.l. (Wharton 1988). Hosts are unknown for nearly all of these, but at least one of the species is recorded from tephritids (Costa Lima 1938). Most species of *Diachasmimorpha*, another group of tephritid parasitoids, retain the lateral portion of the occipital carina, but there are parallel losses of the carina within Old and New World species groups that have caused confusion in the placement of a few species. *Parasteres* Fischer, for example, was defined solely on the basis of the loss of the occipital carina relative to other species with a short second submarginal cell. *Parasteres* originally included two species, each described from a single male specimen (Fischer 1964, 1967a). The type species of *Parasteres* was subsequently discovered to be the male of the Old World species *Diachasmimorpha tryoni* (Cameron), with the holotype collected during a recovery program in Puerto Rico where *Diachasmimorpha tryoni* had been released for control of tephritid pests. The second species originally included in *Parasteres* is treated below and belongs to the *Diachasmimorpha mexicana* species group, endemic to the New World. The members of the *mexicana* species group are difficult to place because the occipital carina is present as a very short spur ventrally but the spur is easy to overlook and is often obscured by other body parts. Members of the *mexicana* species group have proven challenging to identify because two of the three previously described species were based on single male specimens and female ovipositor length is an important diagnostic feature. *Diachasmimorpha* was not recognized as valid until after publication of Fischer’s (1972, 1977, 1987) monographs of the World Opiinae. Thus, a number of species undoubtedly remain incorrectly placed in other genera and no comprehensive key to species is available (but see Wharton and Yoder 2012).

**Figures 5–8.**
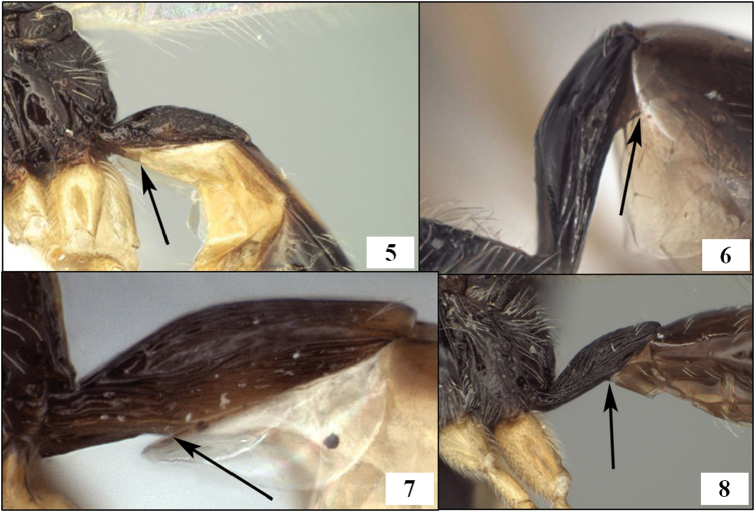
T1 and S1, arrows at posterior margin of S1. **5** *Eurytenes (Stigmatopoea) macrocerus* (Thomson) **6** *Lorenzopius calicomyzae* van Achterberg and Salvo, holotype female **7** *Eurytenes (Stigmatopoea) maya* Wharton sp. n., paratype female **8** *Tubiformopius tubigaster* (Fischer), holotype male.

The first metasomal segment, often referred to as the petiole (Sharkey and Wharton 1997), consists of a heavily sclerotized tergite (T1) and sclerotized sternite (S1) of varying length (Figs 5–8). In several New World species of Opiinae, the petiole is long and more or less parallel-sided. At least two genus-group names have been proposed for species with this characteristic: *Lorenzopius* van Achterberg and Salvo, 1997 and *Tubiformopius* Fischer, 1998. The relationships of the four explicitly included species to others in the Opiinae have not been discussed previously, nor is it clear that the feature used to define these two taxa (an elongate, tube-shaped T1) is sufficiently characterized to enable assessment of homology across the various species with an elongate petiole. Walker and Wharton (2011), for example, described a new species of *Eurytenes* s.s. with an exceptionally long, tubular petiole and Wharton (1988) placed *Opius macrocerus* Thomson in *Eurytenes* partly on the basis of a narrow, parallel-sided petiole.

Van Achterberg and Salvo (1997) described *Lorenzopius* and characterized it on the basis of the tube-shaped petiole (Fig. 6) with at least the basal half of the tergite closed ventrally and with a midpit on the mesoscutum posteriorly. Three species were originally included: *Opius tubulatus* Fischer, 1979, *Opius sanlorenzensis* Fischer, 1964, and the type species, *Lorenzopius calycomyzae* van Achterberg and Salvo. *Opius tubibasis* Fischer, 1978 was also mentioned as a potential member of the newly described genus. Almost concurrently, Fischer (1998) described *Tubiformopius*, which he later (Fischer 1999) treated as a synonym of *Lorenzopius*. However, the type species of *Tubiformopius* (*Opius tubigaster* Fischer, 1968), while possessing a tubular petiole (Fig. 8), differs from *Lorenzopius calycomyzae*, *Lorenzopius tubulatus*, and *Lorenzopius sanlorenzensis* in several important aspects. In *Tubiformopius tubigaster*, there is no midpit on the mesoscutum, fore wing m-cu is widely antefurcal, the first subdiscal cell is broadly open distally, and the mandible has a distinct basal lobe (= basal tooth). Given these differences, I retain *Tubiformopius* as valid, at least for the present, and also include *Tubiformopius tubibasis*, new combination, since it shares these and other features with *Tubiformopius tubigaster*.

Neither van Achterberg and Salvo (1997) nor Fischer (1998) mentioned the sternite in their descriptions, focusing instead on the tubular tergite, closed ventrally. What is most distinctive about *Lorenzopius* and *Tubiformopius*, however, is the length of S1 and its apparent fusion with T1. The presence of a prominent S1 is an unusual feature in the Opiinae and it is therefore not surprising that two genus group names have been proposed for species with this characteristic. In the vast majority of opiine species S1 is present as a very short basal sclerite, clearly separated by membrane from the tergite, but S1 is often overlooked because it is difficult to see without removal of at least one hind leg. The type species of *Tubiformopius* and *Lorenzopius* have sternites illustrating different positions along the morphocline of an increasingly elongate S1 that appears fused to the tergite. S1 in *Tubiformopius tubigaster* is 0.5–0.6 times the length of T1 (Fig. 8) while S1 in *Lorenzopius calycomyzae* and *Lorenzopius tubulatus* extends nearly the full length of T1 (Fig. 6). The other differences noted above between the type species of *Tubiformopius* and *Lorenzopius* make it relatively easy to place *tubibasis* in *Tubiformopius* rather than *Lorenzopius*, but other species with a narrow T1 and an elongate S1 are more challenging to place. Two such examples, *Opius incoligma* Fischer and *Opius rugicoxis* Fischer (Figs 74–83) are diagnosed below under *Opius* to highlight the problems in placing such species when focusing only on the presence of an elongate S1. *Eurytenes* is also problematic since several species have long, narrow petioles. The species of *Lorenzopius* are similar in many respects to *Eurytenes macrocerus* (Thomson), the type species of *Eurytenes* (*Stigmatopoea*). Both have an exposed labrum with sharp ventral margin to the clypeus, relatively well-developed notauli, a distinct midpit on the mesoscutum, relatively large scuto-scutellar sulcus, and similar venation, most notably the parallel-sided stigma. In *Stigmatopoea*, however, the dorsope is retained and S1, though longer than in most Opiinae, is short relative to *Lorenzopius* and clearly separated from T1 (as in Fig. 5). The number of shared features may indicate that *Lorenzopius* represents a distinct lineage derived from a *Stigmatopoea*-like ancestor. Otherwise, the exceptional morphological diversity in the species that share an elongate S1 suggests homoplasy, with possibly multiple derivations of an elongate S1. Until the relationships among the many Neotropical species with an elongate S1 are better understood, this feature will remain useful for characterizing opiine species, but must be used cautiously and in combination with other characters for defining genera.

## Taxonomy

### Diachasmimorpha

Viereck

http://species-id.net/wiki/Diachasmimorpha

Diachasmimorpha Viereck, 1913: 641. Type species: *Diachasmimorpha comperei* Viereck, 1913 [a junior subjective synonym of *Diachasmimorpha longicaudata* (Ashmead, 1905)]. Monobasic and original designation.
Biosteres (*Parasteres*) Fischer, 1967a: 3. Type species: *Biosteres (Parasteres) acidusae* Fischer, 1967a [a junior subjective synonym of *Diachasmimorpha tryoni* (Cameron, 1911)]. Original designation.
Parasteres : Fischer 1971: 33 (change in rank). Synonymized under *Biosteres* by Wharton and Marsh (1978:154) and under *Diachasmimorpha* by Wharton (1987a: 62).

#### Diagnosis.

Mandible without basal lobe ventrally. Labrum concealed. Occipital carina broadly absent dorsally, present or absent laterally. Propleuron ventral-laterally without oblique carina. Notauli deep, unsculptured or nearly so, well developed anteriorly, varying posteriorly from absent to deep and complete to midpit; midpit always present. Fore wing stigma short, broad, discrete posteriorly, r1 arising at or distad its midpoint; second submarginal cell short; m-cu arising from second submarginal cell. Hind wing RS absent basally, sometimes present as a weakly pigmented crease distally; 2M distinctly pigmented nearly to wing margin; m-cu present, well-developed. Dorsope absent.

**Figures 9–12.**
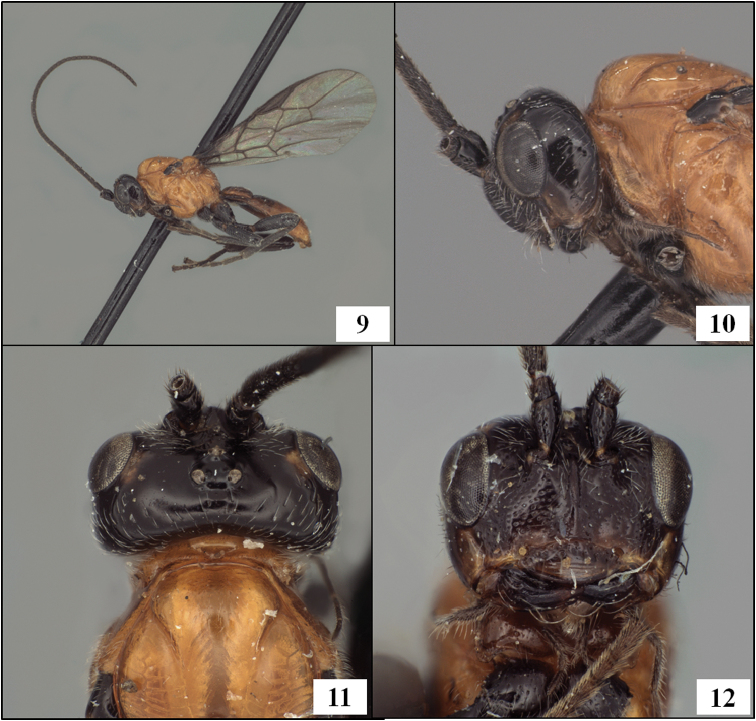
*Diachasmimorpha hildagensis* (Fischer), holotype male. **9** habitus **10** head and base of notaulus, lateral view **11** head, pronope, and base of notaulus, dorsal view **12** face.

The species of *Diachasmimorpha* are most readily recognized by the pattern of fore and hind wing venation (Figs 9, 16) in combination with the concealed labrum (Fig. 12), unsculptured notauli (Figs 11, 14, 19, 20), and lack of oblique carina on the propleuron (Fig. 23). The species of *Doryctobracon* Enderlein, endemic to the New World, are similar but have the fore wing m-cu interstitial or arising from the first submarginal cell and the labrum is partially exposed. *Fopius* Wharton, an Old World genus with species that have been introduced to the New World, is also similar. The species of *Fopius* differ by the presence of completely sculptured notauli and the presence of an oblique carina on the propleuron (Fig. 24).

#### Remarks.

Both New and Old World species groups of *Diachasmimorpha* occur in Mexico. *Diachasmimorpha longicaudata* (Ashmead) and *Diachasmimorpha tryoni*, both representatives of the Old World *longicaudata* species group (Wharton 1997), were established in various parts of Mexico during biological control programs directed against tephritid pests primarily in the genus *Anastrepha*. Females of the Old World species are readily distinguished from New World *Diachasmimorpha* because of the sinuate ovipositor (Fig. 28). The notauli are also more deeply incised posteriorly in the *longicaudata* species group (Fig. 19, in contrast to Fig. 20), which facilitates identification of males in biological control and other tephritid pest management programs. The name *Parasteres* continues to be used by some authors, for example as a subgenus of *Diachasmimorpha* (Yu et al. 2012), but we continue to treat *Diachasmimorpha tryoni* and *Diachasmimorpha longicaudata* in the same species group based in part on ovipositor morphology. We therefore do not treat *Parasteres* as valid, nor do we recognize subgenera under *Diachasmimorpha* at this time.

New World species have previously been referred to as the *mexicana* species group (Wharton 1997), a use we continue here. Wharton (1997) noted, however, that there were two subgroups distinguished in part on the basis of relative loss of the occipital carina. Further examination and discovery of additional species provides support for the two subgroups. One of these subgroups consists of *Diachasmimorpha juglandis* (Muesebeck), *Diachasmimorpha mellea* (Gahan), and *Diachasmimorpha sublaevis* (Wharton). The occipital carina is generally better developed in this subgroup (usually readily visible laterally as in Fig. 2), the wings are hyaline, and the body is yellowish. As in the *longicaudata* species group, the anterior margin of the pronotum ventral-laterally is sharply excavated (Fig. 17). The second subgroup contains *Diachasmimorpha mexicana* (Cameron), *Diachasmimorpha sanguinea* (Ashmead), *Diachasmimorpha hildagensis* (Fischer), new combination, and the new species described below. In all of these species, the occipital carina is greatly reduced, present only as a short spur ventrally near the mandible (maximum extent shown in Fig. 4). These species also have infumate wings (Fig. 16) and the body tends to be orange rather than yellow. The anterior margin of the pronotum ventral-laterally is also more sinuate than abruptly excavated (Fig. 18). Detailed diagnoses are provided below for the three previously described species in this second subgroup, to facilitate comparison with the newly described species.

#### Key to species of *Diachasmimorpha* known from U.S. and Mexico

**Table:** 

1	Female (ovipositor clearly visible, extending well beyond apex of metasoma)	2
–	Male	10
2 (1)	Ovipositor distinctly sinuate subapically (Fig. 28)	3
–	Ovipositor straight or nearly so subapically (Fig. 29)	4
3 (2)	Metasomal tergum 2 distinctly striate medially (Fig. 21). Occipital carina well developed laterally, extending from base of mandible at least to mid eye height	*Diachasmimorpha longicaudata* (Ashmead)
–	Metasomal tergum 2 without striae or other sculpture (Fig. 22). Occipital carina poorly developed to absent, not extending dorsally to lower eye margin	*Diachasmimorpha tryoni* (Cameron)
4 (2)	Head dark, at least on dorsal half (Fig. 9)	5
–	Head pale (Figs 2, 4), yellow or orange except sometimes ocellar field dark	7
5 (4)	Ovipositor (total length) about 2.5 times longer than mesosoma. Notaulus extending anteriorly to margin of mesoscutum (Figs 10, 27)	6
–	Ovipositor (total length) less than 2.0 times longer than mesosoma. Notaulus rarely extending anteriorly to margin of mesoscutum, usually terminating just before reaching margin (Fig. 32)	*Diachasmimorpha norrbomi*, sp. n.
6 (5)	Eye smaller than in Fig. 32, about 1.5–1.6 × longer than temple in lateral view	*Diachasmimorpha hildagensis* (Fischer)
–	Eye larger, 2.1–2.9 × longer than temple in lateral view (Fig. 33)	*Diachasmimorpha martinalujai*, sp. n.
7 (4)	Wings darkly infumate (as in Figs 16, 36). Occipital carina represented at most as in Fig 4, usually present as a short spur near mandible, otherwise absent	*Diachasmimorpha sanguinea* (Ashmead)
	Note: *mexicana* (Cameron) also keys here but is known only from the male, which has a much smaller eye than that of *sanguinea*.
–	Wings hyaline (Fig. 20). Occipital carina present laterally at least to lower margin of eye, usually as in Fig. 2	8
8 (7, 14)	Metasomal tergum 2 distinctly striate medially (as in Fig. 21)	9
–	Metasomal tergum 2 without striae or other sculpture (as in Fig. 22)	*Diachasmimorpha juglandis* (Muesebeck)
9 (8)	Precoxal sulcus distinctly impressed, usually broad but very weakly sculptured, nearly smooth (as in Fig. 38). Hosts are walnut husk flies in species of *Juglans D. sublaevis* (Wharton)
–	Precoxal sulcus distinctly impressed, broad, heavily sculptured: crenulate to foveolate (as in Fig. 17). Hosts are other species of *Rhagoletis* in other fruits	*Diachasmimorpha mellea* (Gahan)
10 (1)	Head black at least over dorsal half	11
–	Head pale, yellow to orange except ocellar triangle sometimes black	13
11 (10)	Eye in dorsal view as long as temple; eye in lateral view 1.3–1.4 × longer than temple	*Diachasmimorpha hildagensis* (Fischer)
–	Eye slightly larger, in dorsal view eye 1.4–1.9 × longer than temple, in lateral view 1.7–2.4 × longer than temple	12
12 (11)	Notaulus extending anteriorly to margin of mesoscutum (Fig. 27)	*Diachasmimorpha martinalujai*, sp. n.
–	Notaulus rarely extending anteriorly to margin of mesoscutum, usually terminating just before reaching margin (Fig. 32)	*Diachasmimorpha norrbomi*, sp. n.
13 (10)	Metasomal tergum 2 striate medially (Fig. 21)	14
–	Metasomal tergum 2 without striae or other sculpture (Fig. 22)	15
14 (13)	Notauli deep posteriorly as it nears midpit (Fig. 19)	*Diachasmimorpha longicaudata* (Ashmead)
–	Notauli more shallow posteriorly as it nears midpit (Fig. 20)	8
15 (13)	Metasomal terga mostly black (Fig. 22)	*Diachasmimorpha tryoni* (Cameron)
–	Metasoma with at least terga 3–5 pale: yellow to orange	16
16 (15)	Wings hyaline	*Diachasmimorpha juglandis* (Muesebeck)
–	Wings darkly infumate	17
17 (16)	Eye larger, about 1.3–1.5 × longer than temple in lateral view	*Diachasmimorpha sanguinea* (Ashmead)
–	Eye smaller, subequal to temple in lateral view (Fig. 35)	*Diachasmimorpha mexicana* (Cameron)

### Diachasmimorpha hildagensis

(Fischer) comb. n.

http://species-id.net/wiki/Diachasmimorpha_hildagensis

Figs 9–12 13–16

Opius (Biosteres) hildagensis Fischer, 1964: 12, 20–22. Holotype male in AEIC (examined).
Biosteres (Parasteres) hildagensis : Fischer 1967a: 5 (generic transfer).
Parasteres hildagensis : Fischer 1971: 33 (generic transfer); Fischer 1977: 880–883 (key, redescription).

#### Type locality:

Mexico, State of Mexico, Hidalgo National Park.

#### Type material.

Holotype male (AEIC), first label, first line: Hidalgo Natl. Pk. second line: State of Mex., Mex. third line: x.12.62 3000 m. fourth line: H. & M. Townes Second label [purple]: Holotype Third label: Opius hildagensis [male symbol] sp. n. det. Fischer Fourth label: Type No. 336

**Other specimens examined:** 2 females, 1 male, Mexico, Mexico, Rt 890, km 9, 6 km W Lago Zempoala, 2.x.1991, A.L. Norrbom, reared from *Oedicarina latifrons* infesting fruits of *Solanum brachycarpum* (91M14B) (TAMU, USNM).

#### Diagnosis.

Holotype male. Eye in dorsal view as long as temple, temples neither receding nor expanded beyond eyes; eye in lateral view 1.3 × longer than temple. Frons irregularly rugulose along midline between antenna and median ocellus. Clypeus 2.8 × wider than high. Occipital carina distinct near base of mandible, short, not extending dorsally to ventral margin of eye. Antenna with 46 flagellomeres; first flagellomere 1.25 × longer than wide. Pronope deep, large, interrupting posterior crenulate groove middorsally. Notauli deep anteriorly, reaching anterior-lateral margin of mesoscutum and extending posteriorly about 0.5 × distance to deep, elongate midpit. Precoxal sulcus distinctly crenulate throughout, nearly extending to anterior margin of mesopleuron. Propodeum rugose, areola extending over posterior 0.6 but largely obscured by sculpture. Fore wing 2RS 0.95 × length of 3RSa; m-cu distinctly postfurcal. T1 with dorsal carinae weakly converging, widely separated at posterior margin, gradually weakening posteriorly. Meso- and metasoma orange, tegula black, head dark brown to black except narrow yellow-orange band along epistomal sulcus extending to and through malar sulcus and small orange spot on vertex adjacent eye; legs black except extreme base of hind coxa irregularly orange, joint between femora and trochantelli reddish orange, mid and hind tarsi dark brown. Body length about 4.3 mm, fore wing length 4.5 mm, mesosoma length 1.8 mm.

Specimens reared from *Oedicarena latifrons* (Wulp) vary as follows relative to the holotype: clypeus length/height ratio 2.6–2.8; eye/temple ratio, lateral view, 1.3–1.4 (males), 1.55 (female); antenna with 46–48 flagellomeres; 2RS/3RS ratio 0.95–1.0; ovipositor sheath 2.5 times longer than the mesosoma; mesosoma length 1.85–1.9 mm (male), 2.0 mm (female); one male with T1 dorsal carinae absent over posterior 0.5 and mandible, clypeus, face, and hind coxa more extensively orange; female with outer surface of hind coxa completely pale (dark medially), mandible, clypeus and lower part of face more extensively pale than in holotype.

This species is slightly larger and has a smaller eye than both of the similarly-colored species described below, *Diachasmimorpha martinalujai*, sp. n. and *Diachasmimorpha norrbomi*, sp. n. Based on the single female reared from *Diachasmimorpha latifrons*, *Diachasmimorpha hildagensis* alsohas a much longer ovipositor than *Diachasmimorpha norrbomi*. The ovipositors of *Diachasmimorpha hildagensis* and *Diachasmimorpha martinalujai* are similar in length. In *Diachasmimorpha hildagensis* and *Diachasmimorpha martinalujai*, the notaulus consistently extends anteriorly to the margin of the mesoscutum whereas in *Diachasmimorpha norrbomi*, the notaulus usually does not. Color variation in the specimens reared from *Opius latifrons* is similar to that in the paratype series of *Diachasmimorpha martinalujai* and *Diachasmimorpha norrbomi*. Both *Diachasmimorpha hildagensis* and the two newly described speciesare similar in having the head mostly dark in contrast to the orange heads of *Diachasmimorpha mexicana* and *Diachasmimorpha sanguinea*, the other two members of this species group. The holotype of *Diachasmimorpha hildagensis* exhibits subsurface discoloration on the metasoma, but the tergites are all entirely orange.

#### Biology.

There is no biological information associated with the holotype. The non-type material listed above was reared from the tephritid *Oedicarina latifrons* infesting fruits of *Solanum brachycarpum* Correll. Collection data and host information can be found in Norrbom et al. (1988).

#### Remarks. 

**Figures 13–16.**
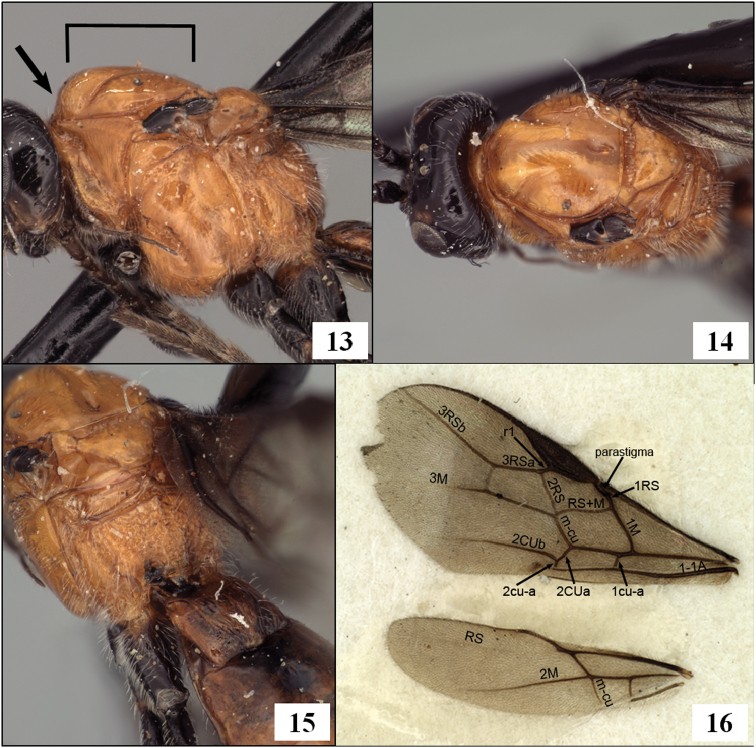
*Diachasmimorpha hildagensis* (Fischer), holotype male. **13** mesosoma, lateral view, arrow showing anterior declivity of mesoscutum, bracket showing mesoscutal disc **14** head and mesonotum, dorsal view **15** propodeal sculpture **16** left fore and hind wings illustrating wing vein terminology.

The name *hildagensis* is based on a misreading of the locality label on the holotype, which is correctly written as Hidalgo Nat. Park, not “Hildago Nat. Park” as given by Fischer (1964) in the original description. In the original description, *hildagensis* is included in a key to the subgenus *Biosteres*, but the subgeneric name was not included in the heading for the species description. This species is here transferred to *Diachasmimorpha*, as diagnosed above, on the basis of fore and hind wing venation (Fig. 16), the morphology of the labrum, clypeus, and mandible (Fig. 12), and the well-developed notaulus and midpit (Figs 13–15). A detailed description of *Diachasmimorpha* is provided in Wharton (1997). Inclusion of *Diachasmimorpha hildagensis* in the *mexicana* species group is based on the greatly reduced occipital carina, sinuate anterior margin of the pronotum ventral-laterally, and the body and wing coloration.

Both *Diachasmimorpha hildagensis* and *Diachasmimorpha mexicana* were described from single male specimens collected in the state of Mexico and the Distrito Federal, respectively, and unassociated with either hosts or host plants. Both have relatively small eyes, but are readily separated from one another on the basis of head coloration. Associating the name *hildagensis* with the many dark-headed specimens available for study, however, has been considerably more challenging. Reared material, representing over 50 specimens kindly made available to us by Allen Norrbom, Martin Aluja, and Juan Rull, provides clear evidence of sexual dimorphism in eye size as well as variation in ovipositor length associated with different hosts and host plants. This material has been especially critical for understanding color patterns and associating males with females. Based primarily on eye size and body size, the holotype of *Diachasmimorpha hildagensis* is closest to the series of three specimens listed above under “other specimens examined,” that emerged from puparia of *Opius latifrons* infesting fruits of *Solanum brachycarpum*. From the remaining reared material, we describe two closely similar species below.

### Diachasmimorpha martinalujai

Wharton sp. n.

urn:lsid:zoobank.org:act:9E85B215-4032-4CEC-ADF4-6F068E83C029

http://species-id.net/wiki/Diachasmimorpha_martinalujai

Figs 26, 27 31, 33

#### Type locality.

Mexico, Distrito Federal.

#### Type material.

Holotype. Female (UNAM), first and only data label, first line: Mexico, D. F. second line: Host = R. pomonella third line: Host plant=Crataegus sp. fourth line: Common name=Tejocote fifth line: 7.xi.2007 J. Rull

**Paratypes:** 1 male, same data as holotype (TAMU). 1 male, Mexico, Hidalgo, Atotonilco, 4.xi.2002, J. Rull, key 30, reared from *Rhagoletis* nr. *pomonella* infesting fruit of *Crataegus* spp. (TAMU). 1 male, Mexico, Puebla, San Martin, 24.xi.2003, M. Pale key 69, reared from *Rhagoletis* nr. *pomonella* infesting fruit of *Crataegus mexicana* (TAMU).

#### Description.

*Female*.Head in dorsal view 1.30 × broader than mesoscutum, 1.65 × broader than face; eye in dorsal view 2.0 × longer than temple, temples not receding, but width at eyes greater than width at temples; eye in lateral view 2.05 × longer than temple. Discrete facial midridge ending dorsally as a distinct elevation at base of antennae, continuing between antennae onto frons as low, sharp, bifurcating ridges. Frons irregularly rugulose along midline between bifurcating arms, otherwise polished, with moderately dense patch of decumbent, laterally-directed, white setae on either side of midline; bare on either side of ocellar field; width of ocellar field 0.95 × distance from ocellar field to eye. Face 2.2 × wider than high; uniformly setose (as in Figs 31, 33), distinctly punctate, punctures separated by about 1 × their diameter or slightly less. Malar sulcus deep, complete; malar space about 1.1 × basal width of mandible, 0.35 × eye height. Clypeus 2.65 × wider than high; very weakly convex, nearly flat. Occipital carina weak, difficult to discern near base of mandible, short, extending dorsally to ventral margin of eye. Hypostomal carina extending as short but distinct flange below mandible. Antenna with 45 flagellomeres; first flagellomere 1.3 × longer than second; 1.8 × longer than wide.

Mesosoma 1.4 × longer than high; 1.9 × longer than wide; 1.35 × higher than wide. Pronotum not visible dorsally; crenulae extending over dorsal 0.3–0.4 of pronotum laterally within narrow, shallow groove; groove not margined anteriorly by carina; anterior margin of pronotum laterally sinuate, not abruptly excavated. Notauli deep anteriorly, ending abruptly posteriorly, short, not quite extending posteriorly to level of anterior margin of tegula, not reaching long, narrow midpit, anterior end extending to anterior-lateral margin of scutum; mesoscutum without supra-marginal carina adjacent margin of mesoscutum between base of notaulus and tegula. Scuto-scutellar sulcus rectangular or nearly so; 4.75 × wider than midlength; crenulate-foveolate. Propodeum rugose, areola extending over posterior 0.8 but partially obscured by sculpture. Precoxal sulcus crenulate, distinctly separated from anterior margin of mesopleuron.

Wings. Fore wing stigma short, broad, discrete distally, 3.5 × longer than wide; r1 arising from midlength of stigma; 1RS (excluding parastigma) 0.30 × length of 1M; m-cu postfurcal by 0.25 × length of m-cu; second submarginal cell converging distally; 2RS 0.9 × length of 3RSa; 2CUa about 1.7 × longer than 2cu-a; 1cu-a distad 1M by about 1.0 × its length.

Metasoma not distinctly petiolate; head 1.8 × wider than apex of T1. T1 1.05 × as long as apical width; strongly diverging apically, with apex 2.1 × wider than base; surface smooth; dorsal carinae parallel-sided, widely separated posteriorly, distinctly elevated over anterior 0.6, weaker and becoming indistinct posteriorly; lateral carina weaker than dorsal carina basally, extending distinctly ventrad spiracle, rounded and barely distinguishable posteriorad spiracle; spiracle at midlength of T1; dorsope absent but lateral and dorsal carinae elevated at junction, giving appearance of a slight depression; laterope deep; S1 very short. T2 unsculptured, with sharp lateral margins. Ovipositor sheath 2.4 × longer than mesosoma, densely setose over apical half, with 4–5 irregular rows of setae, the setae longer than sheath width, more sparsely setose basally.

Color (Fig. 26). Very similar to *Diachasmimorpha hildagensis*. Meso- and metasoma orange, except tegula black; head dorsally black except for small orange spot on vertex adjacent eye; lower gena and most of occiput yellow-orange; narrow bands dorsad epistomal sulcus, along ventral margin of clypeus and vertically through middle of mandible orange; legs black to dark reddish brown except basal 0.5 of hind coxa orange, joint between femora and trochantelli reddish orange.

*Male*. Largely as in female with variation as follows: head in dorsal view 1.35–1.45 × broader than mesoscutum, 1.6–1.7 × broader than face; eye in dorsal view 1.6–1.85 × longer than temple, in lateral view 1.7–1.95 × longer than temple; face 1.95–2.1 × wider than high; malar space 0.3–0.45 × eye height; clypeus 2.6–2.8 × wider than high; antenna with 39–47 flagellomeres; first flagellomere 1.1–1.2 × longer than second, 2.0–2.1 × longer than wide; mesosoma 1.25–1.35 × longer than high; 1.85–1.95 × longer than wide; 1.4–1.5 × higher than wide; pronope deep, moderately large but not interrupting posterior crenulate groove middorsally; crenulae extending over dorsal 0.2–0.4 of pronotum laterally; scuto-scutellar sulcus 4.0–5.0 × wider than midlength; areola of propodeum variably obscured, short and triangular rather than pentagonal in topotypic paratype; precoxal sulcus occasionally extending to anterior margin of mesopleuron; fore wing stigma 3.3–3.8 × longer than wide; 1RS 0.2–0.25 × length of 1M; m-cu postfurcal by 0.15–2.0 × length of m-cu; 2RS 0.8–1.05 × length of 3RSa; head 1.85–2.2 × wider than apex of T1; T1 0.95–1.05 × as long as apical width, apex 2.1–2.25 × wider than base; surface of T1 between dorsal carinae weakly rugulose; dorsal carinae weakly sinuate, weakly converging at posterior margin of T1; S1 extending posteriorly only to level of dorsal tendon attachment; head varying from darker as in female to more extensively pale (as in Fig. 31) with ventral 0.5 of face orange, outer surface of mandible entirely dark orange and clypeus reddish brown; hind coxa varying from almost entirely orange to almost entirely black; hind femur and tibia varying from black to reddish brown.

Body length 4.9 mm (female), 3.1–4.7 mm (male), fore wing length 4.0 mm (female), 2.7–4.1 mm (male), mesosomal length 1.55 mm (female), 1.0–1.7 mm (male).

#### Diagnosis.

This species is nearly identical to *Diachasmimorpha hildagensis* based on the similarly long ovipositor and the notaulus that consistently extends all the way to the anterior margin of the mesoscutum. The eye is distinctly larger in *Diachasmimorpha martinalujai* than in *Diachasmimorpha hildagensis*. *Diachasmimorpha norrbomi* is also similar, but has a shorter ovipositor and the notaulus only rarely extends anteriorly to the margin of the mesoscutum.

#### Biology.

This is the species that has been referred to as *Diachasmimorpha mexicana* (vide Wharton) in previous publications on parasitoids of *Rhagoletis* Loew in Mexico (e.g. Rull et al. 2009). The holotype and paratypes were all reared from Mexican populations of *Rhagoletis pomonella* infesting fruits of various species of *Crataegus*, including *Crataegus mexicana* DC., as characterized by Xie et al. (2007).

#### Etymology.

This species is named after Martin Aluja in recognition of his many contributions to tephritid biology, particularly in Mexico.

#### Remarks.

The male paratypes, though only three in number, are remarkably variable in size, with larger individuals closely approaching the size of *Diachasmimorpha hildagensis*. Quantitative measures are also highly variable, which is not surprising given the variation in size.

Detailed assessment of the available reared material suggests the presence of a diverse assemblage of *Diachasmimorpha* species in Mexico, associated with different hosts and host plants. The relatively small morphological differences between *Diachasmimorpha hildagensis* and *Diachasmimorpha martinalujai* are consistent among the available material and the differences in host and host plant associations lend support to the recognition of these as separate species.

**Figures 17–20.**
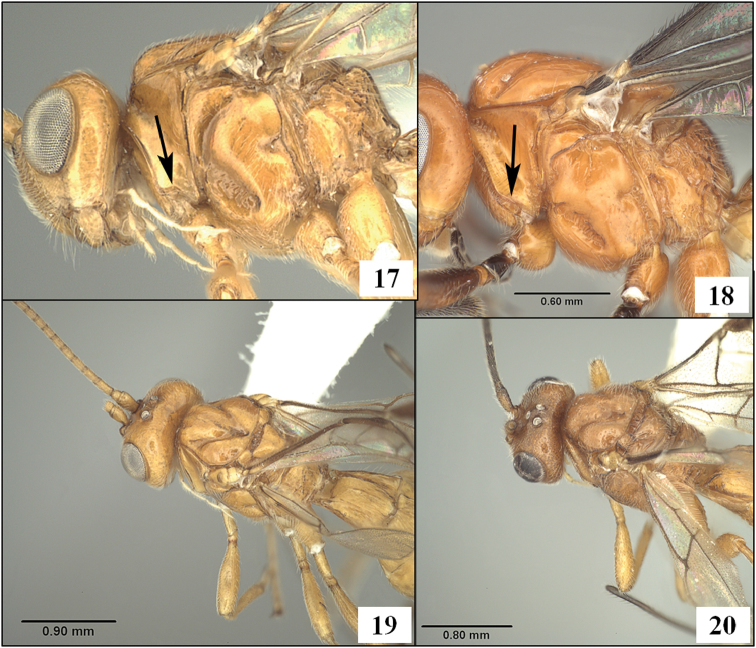
*Diachasmimorpha* spp. **17** *Diachasmimorpha longicaudata* (Ashmead), arrow showing sharply indented margin of pronotum laterally **18** *Diachasmimorpha sanguinea* (Ashmead), arrow showing less sharply indented margin of pronotum laterally **19** *Diachasmimorpha longicaudata*, dorsal view **20** *Diachasmimorpha mellea* (Gahan), dorsal view.

**Figures 21–24.**
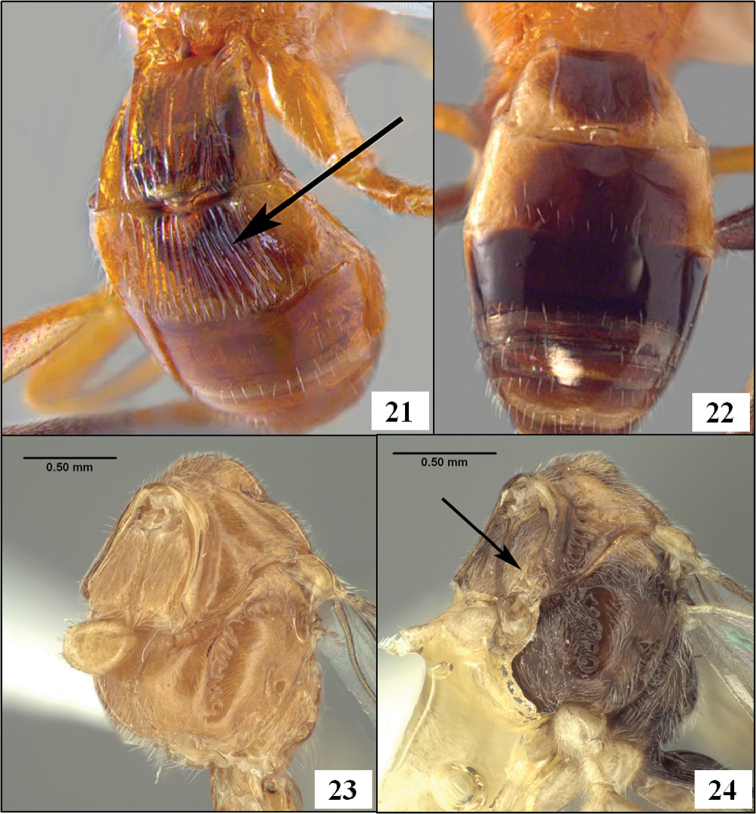
Propleuron and T2. **21** *Diachasmimorpha longicaudata* (Ashmead), T2 with striae (arrow) **22** *Diachasmimorpha tryoni* (Cameron), T2 without sulpture **23** *Diachasmimorpha longicaudata*, propleuron without oblique carina **24** *Fopius arisanus* (Sonan), propleuron with oblique carina (arrow).

### Diachasmimorpha norrbomi

Wharton sp. n.

urn:lsid:zoobank.org:act:4900256F-3E99-41FC-8CCF-3A42E06D2033

http://species-id.net/wiki/Diachasmimorpha_norrbomi

Figs 25, 29 30, 32

#### Type locality.

Mexico, State of Mexico, Parque Lago de Zempoala.

#### Type material.

Holotype. Female (UNAM), first label, first line: Mexico, Parque second line: Lag. de Zempoala, path third line: along L. Zempoala, 10–11. fourth line: VIII.1989, A.L.Norrbom Second label, first line: reared ex. Euphranta second line: mexicana (Tephritidae) third line: ex. fruit of Ribes fourth line: pringlei Rose (89M13)

**Paratypes:** 27 females, 20 males, same data as holotype, one of these with an additional ALN 31 label and a Biosteres sp. 1 det P. Marsh label (TAMU, UNAM, USNM).

Other specimens examined (not paratypes): 1 female, 1 male, Mexico, D.F., Delegacion Tlapan, Fracc. Tlapuente, 19.ix.2003, M. Aluja #50, reared from fruit of Granadilla (TAMU).

#### Description.

*Female*.Head in dorsal view 1.25–1.30 × broader than mesoscutum, 1.80–1.85 × broader than face; eye in dorsal view 1.7–2.0 × longer than temple, temples not receding, but width at eyes greater than width at temples; eye in lateral view 2.1–2.9 × longer than temple. Facial midridge ending dorsally in short, very weak bifurcation between antennae. Frons irregularly rugulose along midline near bifurcation, otherwise polished, with moderately dense patch of decumbent, laterally-directed, white setae on either side of midline; bare on either side of ocellar field; width of ocellar field 1.0–1.2 × distance from ocellar field to eye. Face 1.80–1.95 × wider than high; uniformly setose (as in Figs 30, 32), distinctly punctate, punctures separated by at least 1 × their diameter. Malar sulcus deep, complete; malar space about 0.9–1.0 × basal width of mandible, 0.30–0.35 × eye height. Clypeus 2.8–3.2 × wider than high; very weakly convex, nearly flat. Occipital carina weak but distinct near base of mandible, short, extending dorsally to ventral margin of eye and often slightly beyond, not reaching mid eye height. Hypostomal carina extending as short but distinct flange below mandible. Antenna with 41–47 flagellomeres; first flagellomere 1.05–1.2 × longer than second; 1.8–2.0 × longer than wide.

Mesosoma 1.35–1.45 × longer than high; 1.85–1.95 × longer than wide; 1.35–1.40 × higher than wide. Pronope deep, large, interrupting posterior crenulate groove middorsally; crenulae extending along dorsal 0.2 of pronotum laterally within narrow, shallow groove; groove not margined anteriorly by carina; anterior margin of pronotum laterally sinuate, not abruptly excavated. Notauli deep anteriorly, gradually weakening posteriorly, extending posteriorly to level of tegula, not reaching long, narrow midpit, anterior end usually just short of and only rarely reaching anterior-lateral margin of scutum; mesoscutum usually without supra-marginal carina between base of notaulus and tegula, rarely with short, weak trace of a carina. Scuto-scutellar sulcus nearly rectangular, a little narrower medially; 4.2–4.8 × wider than midlength; crenulate-foveolate. Propodeum rugose, areola extending over posterior 0.8 but largely obscured by sculpture. Precoxal sulcus crenulate, widely separated from anterior margin of mesopleuron.

Wings. Fore wing stigma short, broad, discrete distally, 3.15–3.30 × longer than wide; r1 arising from midlength of stigma; 1RS (excluding parastigma) 0.30–0.35 × length of 1M; m-cu postfurcal by 0.2–0.3 × length of m-cu; second submarginal cell distinctly converging distally; 2RS 1.0–1.2 × longer than 3RSa; 2CUa 1.6–1.8 × longer than 2cu-a; 1cu-a distad 1M by about 1.0 × its length.

Metasoma not distinctly petiolate; head 1.6–1.9 × wider than apex of T1. T1 0.95–1.05 × as long as apical width; strongly diverging apically, with apex 2.0–2.5 × wider than base; surface smooth to weakly strigose posterior-medially, almost completed smooth laterally; dorsal carinae weakly converging, widely separated at posterior margin, strongly elevated over anterior 0.5, gradually weakening posteriorly; lateral carina weaker, extending distinctly ventrad spiracle, rounded and barely distinguishable posteriorad spiracle; spiracle at midlength of T1; dorsope absent but lateral and dorsal carinae elevated at junction, giving appearance of a slight depression; laterope deep; S1 very short, extending posteriorad to level of dorsal tendon attachment. T2 unsculptured, with sharp lateral margins. Ovipositor sheath 1.7–1.8 × longer than mesosoma, setal pattern about as in *Diachasmimorpha martinalujai*, with slightly greater density basally.

**Figures 25–29.**
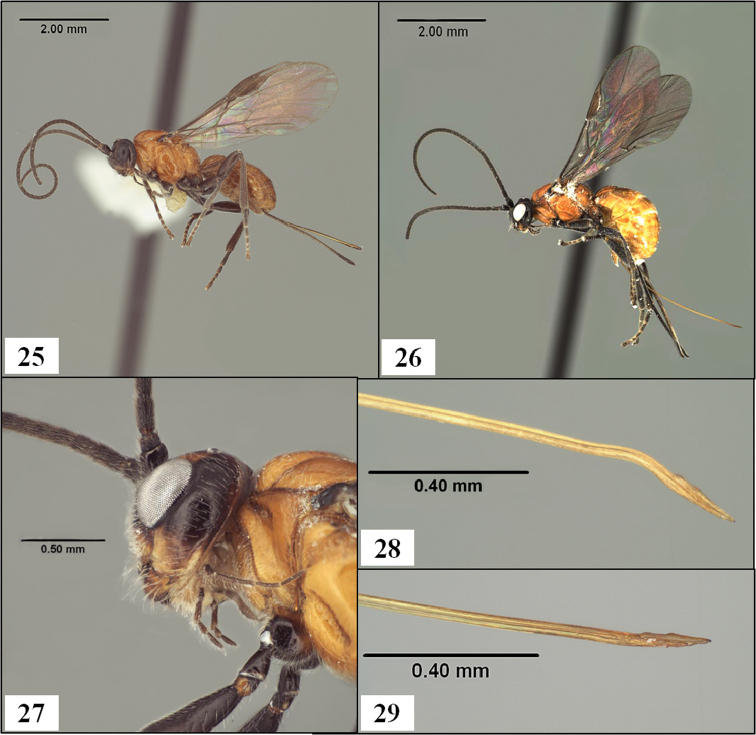
*Diachasmimorpha* spp. **25** *Diachasmimorpha norrbomi* Wharton sp. n., paratype female, habitus showing relatively shorter ovipositor **26** *Diachasmimorpha martinalujai* Wharton sp. n., holotype, habitus showing relatively longer ovipositor **27** *Diachasmimorpha martinalujai* paratype male, base of notaulus **28** *Diachasmimorpha tryoni* (Cameron) apex of ovipositor showing subapical sinuation **29** *Diachasmimorpha norrbomi*, paratype female, apex of ovipositor.

Color (Fig. 25). Very similar to *Diachasmimorpha hildagensis*. Meso- and metasoma orange, except tegula black; head dorsally dark brown to black except for small orange spot on vertex adjacent eye, lower occiput mostly yellow-orange, similar in color to broad band extending through epistomal sulcus, clypeus, lower gena (often), and mandibles; clypeus usually with narrow, transverse brown band, mandible with apical teeth dark, rarely with entire mandible brownish; legs black except extreme base and most or all of dorsal side of hind coxa orange, joint between femora and trochantelli reddish orange.

Male as in female except head in dorsal view 1.3–1.4 × broader than mesoscutum, 1.70–1.75 × broader than face; eye slightly smaller, in dorsal view eye 1.45–1.60 × longer than temple, in lateral view 1.9–2.4 × longer than temple; antenna with 41–43 flagellomeres, first flagellomere 0.95–1.2 × longer than second. Mesosoma slightly narrower, 1.95–2.05 × longer than wide; 1.4–1.5 × higher than wide; scuto-scutellar sulcus somewhat more variable in size, 4.0–5.5 × wider than midlength. Fore wing stigma 3.1–3.4 × longer than wide. T1 slightly smaller, head 1.9–2.2 × wider than apex of T1, T1 1.75–1.90 × wider at apex than at base.

Body length 3.3–4.3 mm, fore wing length 3.5–4.1 mm, mesosoma length 1.15–1.65 mm.

#### Diagnosis.

This species is similar in coloration to *Diachasmimorpha hildagensis* and *Diachasmimorpha martinalujai* but the ovipositor (with sheath 1.7–1.8 × longer than mesosoma) is slightly but distinctly shorter and the notaulus only rarely extends all the way to the anterior margin. The notaulus always reaches the anterior margin in the other two species. *Diachasmimorpha norrbomi* is smaller and has a larger eye than *Diachasmimorpha hildagensis*, and 2RS tends to be longer (relative to 3Ra) in *Diachasmimorpha norrbomi* than in *Diachasmimorpha hildagensis* and *Diachasmimorpha martinalujai*.

#### Biology.

The type series of *Diachasmimorpha norrbomi* was reared from *Euphranta mexicana* Norrbom infesting fruits of *Ribes pringlei* Rose (Norrbom 1993). Two additional specimens that fit within the morphological limits of this species were reared from an unknown tephritid infesting *Passiflora ligularis* Juss.

#### Etymology.

This species is named for Allen Norrbom, who reared many Opiinae from various fruit, stem, and flower-infesting tephritids in Mexico and Central America.

#### Remarks.

Size variation in this species is similar to that exhibited by *Diachasmimorpha martinalujai*, with males dominating the small end of the range.

**Figures 30–33.**
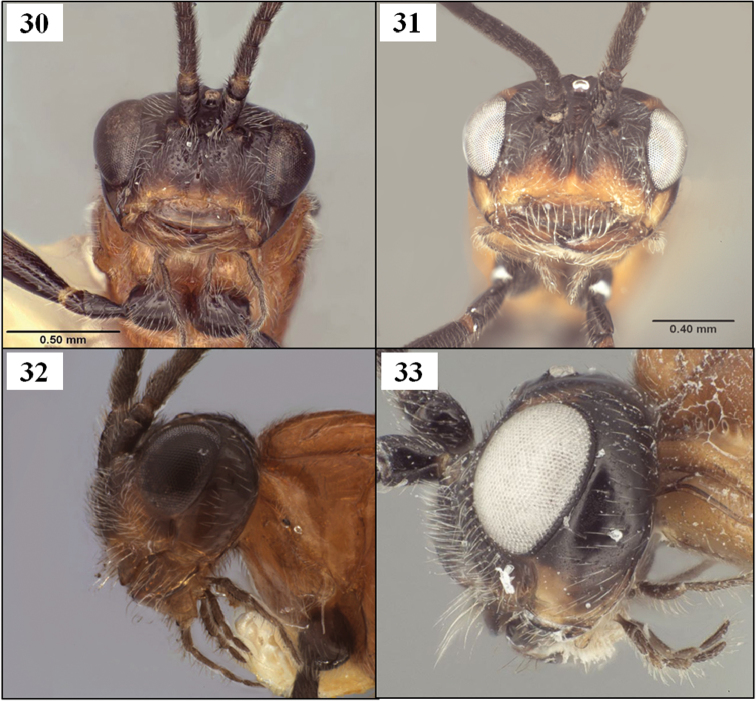
*Diachasmimorpha* spp., heads. **30** *Diachasmimorpha norrbomi* Wharton, sp. n., paratype female, face **31 ***Diachasmimorpha martinalujai* Wharton, sp. n., paratype male, face **32** *Diachasmimorpha norrbomi* paratype female, lateral view **33 ***Diachasmimorpha martinalujai*, holotype female, face.

### Diachasmimorpha mexicana

(Cameron)

http://species-id.net/wiki/Diachasmimorpha_mexicana

Figs 34 38

Opius mexicanus Cameron, 1887: 409–410. Holotype male in BMNH (examined).
Desmiostoma mexicana : Fischer 1967b: 63–64 (redescription, generic transfer); Fischer 1977: 849, 872–873 (key, redescription).
Diachasmimorpha mexicana : Wharton 1997: 14 (generic transfer).

#### Type locality.

Mexico, D. F., Chapultepec.

#### Type material.

Holotype male (BMNH), first label [round, white with red margin], first line: Type second line: H. T. Second label, first line: B. M. TYPE second line: HYM third line: 3.^c.^705 Third label, first line: B.C.A. Hymen. I. second line: Opius third line: mexicanus fourth line: Cam. Fourth label, first line: Opius second line: mexicanus third line: Cam. Type fourth line: BCA ii 409 Fifth label, first line: Bilimek second line: Mexico third line: 1871. fourth line: Chapul fifth line: tepek.

#### Diagnosis.

Holotype male. Eye in dorsal view shorter than temple, temples weakly expanded beyond eyes; eye in lateral view 0.95 × length of temple. Frons unsculptured along midline between antenna and median ocellus. Clypeus 3.4 × wider than high. Occipital carina distinct near base of mandible, short, not extending dorsally to ventral margin of eye. Antenna broken. Pronope deep, large, interrupting posterior crenulate groove middorsally. Notauli deep anteriorly, reaching margin of mesoscutum anteriorly, apparently extending about half distance from anterior-lateral margin to elongate midpit but pin obliterates midpit and surrounding area of mesonotum. Precoxal sulcus very weakly crenulate, nearly smooth, short, not extending close to anterior margin of mesopleuron. Propodeum largely smooth, with rugulose sculpture largely confined to midline, especially around apex, and along border of metapleuron. Fore wing 2RS 0.8 × 3RSa; m-cu distinctly postfurcal. T1 with dorsal carinae widely separated, short, barely extending to level of spiracle, T1 otherwise unsculptured. Head, meso- and metasoma orange, tegula black; legs black as in holotype of *Diachasmimorpha hildagensis*. Body length about 4.0 mm. This species has a much smaller eye (Figs 35, 37) than the similarly-colored *Diachasmimorpha sanguinea* (Fig. 41) and is also less heavily sculptured. Females are unknown.

#### Biology.

Unknown.

#### Remarks.

The body of the *Diachasmimorpha mexicana* holotype is remarkably smooth relative to that of other species in the *mexicana* species group. The precoxal sulcus, for example, is very weakly crenulate, the propodeum is very weakly sculptured in general but completely smooth and polished anterior-laterally, and T1 is unsculptured except for the very short dorsal carinae. Sculpture is variable to some extent in other species of this species group, and thus it would be useful to obtain additional specimens of the true *Diachasmimorpha mexicana* to determine the extent of sculptural variation in this species and ascertain whether reduction in sculpture is a useful diagnostic feature.

Fischer (1967b) noted that the specimen labeled as the type in BMNH is a male, but Cameron (1887) indicated in his original description that he was describing a female. The excellent figure in Cameron (1887) matches the type specimen, providing additional evidence of Cameron’s error (either misinterpretation of the male genitalia as an ovipositor or, more likely given the general quality of Cameron’s early work, a typographical error). The holotype was collected by D. Bilimek in Chapultepec and I have interpreted this as the large park that is now within Mexico City. Fischer (1967b) recorded the type label as type no. 3.c.505, but this is an inadvertent error. The type number for this specimens is 3.c.705.

See additional remarks under *Diachasmimorpha hildagensis* above.

**Figures 34–37.**
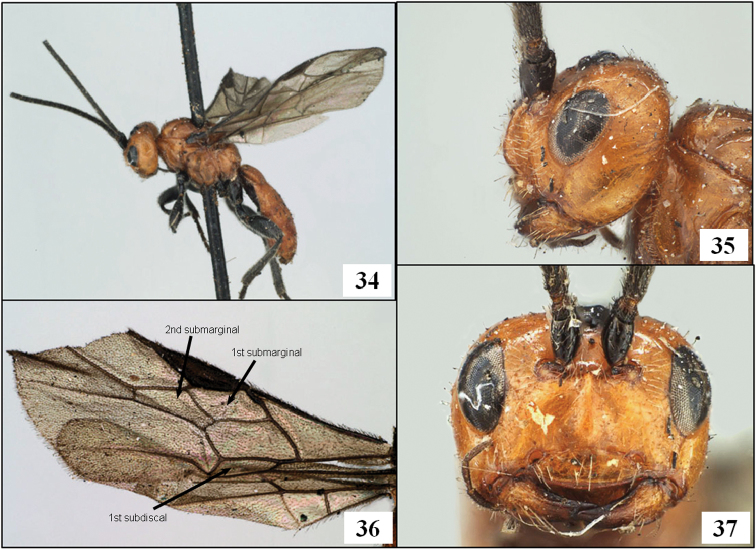
*Diachasmimorpha mexicana* (Cameron), holotype male. **34** habitus **35** head, lateral view **36** wings, showing names of cells used in descriptions **37** face.

**Figures 38–41.**
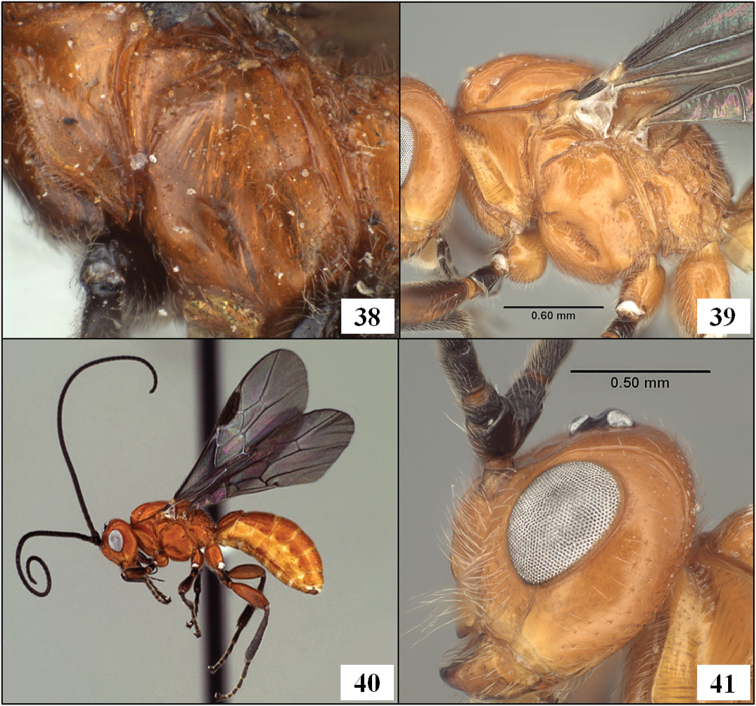
*Diachasmimorpha* spp. **38** *Diachasmimorpha mexicana* (Cameron) holotype male, mesopleuron **39 ***Diachasmimorpha sanguinea* (Ashmead), male mesosoma, lateral view **40** *Diachasmimorpha sanguinea* habitus **41** *Diachasmimorpha sanguinea*, male head, lateral view.

### Diachasmimorpha sanguinea

(Ashmead)

http://species-id.net/wiki/Diachasmimorpha_sanguinea

Figs 4 18 39–41

Phaedrotoma (?) sanguinea Ashmead, 1889: 655. Holotype female in USNM (examined). Marshall 1891: 47 (relationship to a European species of *Opius*).
Opius sanguineus : Gahan 1915: 69, 74 (key, synonymy, expanded distribution and host); Muesebeck and Walkley 1951: 157 (synonymy, new distribution and host); Muesebeck 1967: 54 (catalog).
Opius (Biosteres) sanguineus : Fischer 1965: 116, 138–139 (key, redescription).
Biosteres sanguineus : Fischer 1971: 30 (catalog, change in rank); Wharton and Marsh 1978: 152, 156 (key, diagnosis, distribution, biology); Marsh 1979: 201 (catalog).
Biosteres (Chilotrichia) sanguineus : Fischer 1977: 804, 819–821 (key, redescription).
Diachasmimorpha sanguinea : Wharton 1997: 14 (generic transfer).

#### Type locality.

USA, Washington, D. C.

#### Type material.

Syntype female (USNM), first label, first line: 3737^x^ second line: Oct. 3. 85 Second label (red with black print), first line: Type second line: No2989 third line: U.S.N.M. Third label, first line: Phaedrotoma second line: sanguinea third line: Ashm ms. Syntype male, with same label data as syntype female except Third label = first line: Opius second line: sanguineus third line: Gahan Ashm Syntype male with first label, first line: 3737^x^ second line: Aug. 5. 86 Second label: same as other two syntypes, no third label.

**Other specimens examined.** USA, Texas, 1 female, 1 male, Brazos Co., Yancey, xi.2010, emerged 9.iv & 3.v.2011, L. Ward, reared from *Zonosemata vittigera* infesting fruits of *Solanum eleagnifolium* (TAMU); 1 female, Hidalgo Co., Bentsen Rio Grande Valley State Park, 10.?.1978, C. Porter (TAMU); 5 females, 1 male, Hidalgo Co., Donna, J. W. Monk, reared from *Zonosemata vittigera*; 5 females, 1 male, Jeff Davis Co., 14 mi. S. Ft. Davis, 16–19.viii.1985, L. E. Carroll, reared from *Zonosemata* infesting fruits of *Solanum*; 5 males, Jeff Davis Co., Davis Mts. State Park, 12.vii.1995, R. Wharton; 1 female, Swisher Co., Happy, 17.viii.1977, W. F. Chamberlin.

#### Diagnosis.

Male. Eye in dorsal view 1.1–1.3 × longer than temple, temples not expanded beyond eyes; eye in lateral view 1.3–1.5 × longer than temple. Frons between short, low, bifurcating ridges varying from unsculptured to irregularly strigose, frons otherwise smooth, polished. Clypeus 2.5–2.8 × wider than high. Occipital carina distinct near base of mandible, short, not extending dorsally to ventral margin of eye. Antenna with 38–48 flagellomeres. Pronope deep, large, interrupting posterior crenulate groove middorsally. Notauli deep anteriorly, reaching margin of mesoscutum anteriorly, extending about half distance from anterior-lateral margin to elongate midpit. Precoxal sulcus heavily sculptured, crenulate to foveolate, usually extending to or nearly to anterior margin of mesopleuron. Propodeum rugose, areola, when partially visible, extending over posterior 0.6–0.7 but frequently completely obscured by sculpture. Fore wing 2RS 0.9–1.05 × length of 3RSa; m-cu distinctly postfurcal. T1 with dorsal carinae weakly converging, widely separated at posterior margin, gradually weakening posteriorly, T1 smooth to strigose between carinae. Head, meso- and metasoma orange; tegula orange to brown, legs varying from black except hind coxa mottled black and orange to more extensively orange. Female about as in male except eye in lateral view 1.2–1.6 × longer than temple. Ovipositor sheath 1.6–1.75 × longer than mesosoma. Body length 3.6–5.3 mm, fore wing length 3.3–4.6 mm, mesosoma length 1.2–1.9 mm. This species has a larger eye than the similarly-colored *Diachasmimorpha mexicana* and is generally more heavily sculptured.

#### Biology.

This species was originally described from several specimens reared from a tephritid infesting fruits of *Solanum carolinense* L. (Ashmead 1889). The tephritid host was later identified as *Zonosemata electa* (Say) (Gahan 1915). Muesebeck and Walkley (1951) added *Zonosemata vittigera* (Coquillett) as a host and Cazier (1962) published on the biology of *Zonosemata vittigera* with notes on parasitization by *Diachasmimorpha sanguinea*. The only known host of *Zonosemata vittigera* is *Solanum eleagnifolium* Cav. (Foote et al. 1993) and this is the host plant from which we have reared *Diachasmimorpha sanguinea* in central and western Texas. Adult *Diachasmimorpha sanguinea* are active in summer and fall in Texas, overwinter in the host puparium, and emerge the following year, over a period of several months.

#### Remarks.

The diagnosis is based on the material from Texas listed in the other material examined section. Ashmead (1889) described this species from a single series of reared material, without designation of a type. The specimen in the type collection of the USNM is therefore a syntype, as are the remaining two specimens from this series in the general collection. There is no compelling reason to designate a lectotype, and we have therefore not done so. The original series is currently represented by 2 males and 1 female in the USNM collection. The syntypes agree in all essential details with the material from Texas, though the eye/temple ratio is at the smaller end of the range given above.

The sculpture is somewhat variable in this species, with smaller individuals having a tendency towards rugulose rather than rugose sculpture on the propodeum. The precoxal sulcus is always heavily sculptured, however, never approaching the reduction in sculpture seen in the holotype of *Diachasmimorpha mexicana* (Fig. 39 vs. Fig. 38). The syntypes from Washington, D. C. are as variable in sculpture of the propodeum and T1 as are the specimens from Texas. Specimens from Texas, even within the same reared series, are exceptionally variable in leg coloration. The syntypes from Washington, D. C. have black legs with mostly orange hind coxa. Some specimens from Jeff Davis Co., Texas also have this pattern while in others only the tarsi are dark with the remaining parts orange. Similarly, the tegula is usually orange, but varies from orange to brown even within the same reared series.

*Diachasmimorpha sanguinea* is nearly identical to *Diachasmimorpha mexicana* and additional material from the type locality of the latter is needed for a better understanding of the relationship between these two nominal species.

### Eurytenes

Wesmael

http://species-id.net/wiki/Eurytenes

Eurytenes (*Stigmatopoea* Fischer)
Opius (*Stigmatopoea* Fischer, 1986: 609–611). Type species: *Opius macrocerus* Thomson, 1895. Original designation.
Eurytenes (*Stigmatopoea*): Wharton 1988: 357 (revised status); Fischer 1998: 21–25 (subgeneric keys, diagnoses); Walker and Wharton 2011: 24 (review of classification).
Xynobius (*Stigmatopoea*): van Achterberg 2004: 314–315 (revised status, subgeneric keys).
Eurytenes (*Xynobius*): Wharton 2006: 330–333 (revised status, relationships).

#### Diagnosis.

Mandible without basal lobe ventrally. Labrum broadly exposed. Occipital carina broadly absent dorsally, present laterally. Propleuron ventral-laterally without oblique carina. Notauli deep, well developed anteriorly, varying posteriorly from largely absent to deep and extending to scuto-scutellar sulcus or nearly so; midpit present. Fore wing stigma long, narrow, parallel-sided, discrete posteriorly, r1 arising distinctly basad its midpoint; second submarginal cell with 2RS shorter than 3RSb; 2CUb arising above middle of hind margin of first subdiscal cell. Dorsope present; S1 0.2–0.3 × length of T1, never fused to T1.

#### Remarks.

The new species described below have been placed in *Eurytenes* (*Stigmatopoea*) based on the relative length of S1 (Figs. 5, 7) and the specific characteristics of T1 (Figs 5, 7, 54, 56, 57), wing venation (Fig. 64), mesoscutal sculpture (Figs 44, 48, 49), clypeus (Figs 50–53), and mandibles (Figs 50, 51) listed in the diagnosis. The wing venation is similar to that in *Lorenzopius* but in *Lorenzopius*, the dorsope is absent and S1 is longer and apparently fused to T1 (Fig. 6). We follow Wharton (1988, 2006) and Fischer (1998) in treating *Stigmatopoea* as a subgenus of *Eurytenes*. Wharton (2006) provides a detailed explanation of the morphological basis for this treatment as well as a discussion of alternative classifications.

*Aulonotus* Ashmead has usually been characterized on the basis of well-developed notauli (Fischer 1972, 1998), similar to the condition found in the species described below. *Aulonotus* shares other similarities with *Stigmatopoea*, including the presence of a dorsope, but the petiole is broader, S1 is very poorly developed, the stigma is not parallel-sided, and the precoxal sulcus is distinctly sculptured. Both the type species of *Stigmatopoea* and the two species described here will key to *Opius* (*Nosopoea* Foerster) in Fischer’s classification of Opiinae (Fischer 1972, 1977) because the precoxal sulcus is unsculptured in nearly all individuals (as in Figs 43, 44). Difficulties in interpreting the variable nature of sculpture in the precoxal sulcus, and the emphasis placed on this character in existing keys to Opiinae, make it possible for relatively closely related species to become widely separated in current classifications.

### Eurytenes (Stigmatopoea) maya

Wharton sp. n.

urn:lsid:zoobank.org:act:A5E2449E-78E5-48A3-B4CD-B4FC77A410A4

http://species-id.net/wiki/Eurytenes_maya

Figs 7 42, 44 46, 48 50, 52 56 59 64

#### Type locality.

Mexico, Chiapas, San Cristobal de las Casas.

#### Type material.

Holotype. Female (TAMU), first label, first line: MEXICO: Chiapas second line: San Cristobal de las third line: Casas, xi.2001, #37A fourth line: J. Marquez, M. Aluja Second label, first line: host: Rhagoletis second line: pomonella third line: ex fruit of: fourth line: Crataegus mexicana

**Paratypes:** 2 females, same data as holotype but collected 26.xi.2001, #35A (TAMU); 1 female, same locality, 14.xi.2001, M. Aluja, Key 30A, host: *Rhagoletis* sp. on tejocote, manzanita (TAMU); 1 female, same locality, 14.xi.2001, J. Marquez, ex: *Rhagoletis pomonella* on *Crataegus* sp., #27 (TAMU); 1 female, Chiapas, Rancho Nuevo, 5 km to San Cristobal de las Casas-freeway 190, 15.xi.2002, J. L. Marquez, M. Aluja, # 42, host: *Rhagoletis pomonella* ex fruit of *Crataegus mexicana* (TAMU); 2 males, Chiapas, 3 km E. San Cristobal, 15.xi.1994, R. Jones, ex pupa of *Rhagoletis pomonella* (TAMU); 3 females, Chiapas, Huixtan, 15.ix.2002, J. Marquez, Key 34, host: *Rhagoletis pomonella* ex fruit of *Crataegus* spp. (TAMU); 1 male, 1 female, Chiapas, Cruz Quemada, 15.xi.2002, host: *Rhagoletis pomonella* ex fruit of *Malus* sp., J. Marquez, Key 35, and J. L. Marquez, M. Aluja, #45 (TAMU); 1 male, 1? (abdomen missing), Chiapas, Teopisca, 26.xi.2001, J. L. Marquez, ex: *Rhagoletis pomonella* on *Crataegus* sp. #26 (TAMU).

#### Other specimens examined

(not paratype)**:** 1 male, Mexico: San Luis Potosi, Rio Verde, 7.x.2003, M. Pale, Key 71, *Rhagoletis* nr. *pomonella* on *Crataegus parrayana* (TAMU) [sequenced].

#### Description.

*Female*. Head in dorsal view 1.25–1.30 × broader than mesoscutum, 1.80–1.95 × broader than face; eye in dorsal view 2.5–3.2 × longer than temple, temples distinctly receding behind eyes. Frons and vertex highly polished, unsculptured except for shallow, median depression between toruli; frons bare, vertex and occiput with a few, short, scattered setae; width of ocellar field 1.05–1.3 × distance from ocellar field to eye. Face 1.55–1.70 × wider than high; slightly less polished than frons; uniformly setose (as in Figs 50, 52), with very fine punctures, these separated by at least 2 × their diameter. Frons and face delimited by slight change in sculpture resulting in weak, shallow sulcus between torulus and eye; distance between antennal toruli equal to distance from torulus to eye, eye not distinctly emarginate in region of antenna. Malar sulcus deep, complete; malar space about 0.5 × basal width of mandible, 0.2 × eye height. Face weakly convex, bulging slightly medially along the low midridge. Epistomal sulcus weak mid-dorsally, more distinct laterally. Clypeus 2.2–2.5 × wider than high; weakly convex, slightly protruding in profile; ventral margin sharp, truncate to very weakly concave in frontal view. Labrum broadly exposed, gap between ventral margin of clypeus and dorsal margin of mandible varying from 0.5–1.0 × height of clypeus, depending on how tightly closed the mandibles are. Occipital carina distinctly curved medially at dorsal end, broadly absent mid-dorsally, the space where the carina is absent distinctly wider than width of ocellar field; occipital and hypostomal carinae widely separated at base of mandible, the latter extending as a flange beneath about basal 0.2 of mandible. Mandible without basal lobe ventrally; bidentate apically, lower tooth much smaller than dorsal tooth and slightly twisted beneath dorsal tooth; ventral margin carinate throughout. Antenna 1.35–1.45 ×longer than fore wing, with 39–43 flagellomeres; first flagellomere 1.1–1.3 × longer than second, 1.2–1.3 × longer than third; flagellomeres 2.3–2.7 × longer than wide basally, twice longer than wide apically. Maxillary palps a little longer than head height; fifth and sixth segments equal in length or nearly so, fourth segment 1.1–1.15 × longer than both fifth and sixth.

Mesosoma 1.4 × longer than high; 1.9 × longer than wide; 1.35–1.40 × higher than wide. Pronotum dorsally a narrow, polished, smooth band with crenulate groove along posterior margin; rarely with discernible, slightly enlarged pit in middle of crenulate groove; crenulae extending in narrow, shallow groove onto pronotum laterally, but only covering dorsal 0.2–0.4; groove margined anteriorly by sharp carina that continues ventrally along full length of pronotum. Anterior declivity of mesoscutum completely vertical, bare or nearly so; anterior-lateral corners of mesoscutum at upper edge of declivity elevated, rounded, sparsely setose; notauli extending 0.4 × distance from anterior declivity to scuto-scutellar sulcus, extending posteriorly from lateral side of elevated anterior-lateral corners, not extending to mesoscutal margin anteriorly, very weakly converging posteriorly; narrow, crenulate throughout; mesoscutum with distinct supra-marginal carina extending from elevated anterior-lateral corner to tegula. Lateral and median mesoscutal lobes bare except scattered setae along notauli; midpit deep, round to somewhat elongate, never extending to notauli. Scuto-scutellar sulcus nearly rectangular, a little narrower medially; 3.75–4.25 × wider than midlength; crenulate-foveolate, with 7 ridges; all sides vertical, clearly delineated. Scutellum very weakly convex, nearly flat, not strongly elevated; bare except for scattered setae posteriorly; unsculptured, even along posterior margin. Propodeum with median carina over anterior 0.3, bifurcating at this point to form an inverted v-shaped transverse carina extending to pleural carina just posteriad spiracle; pleural carina complete from base to apex though sometimes partly obscured by sculpture posteriad spiracle; lateral longitudinal carina parallel to and narrowly separated from pleural carina anteriad spiracle, more medially displaced when visible posteriad transverse carinae, forming part of broad areola; area between pleural and lateral longitudinal carinae rugose and sparsely setose anteriorly; lateral propodeal areas anteriorly on either side of median carina smooth, bare, unsculptured; areola broad, varying from distinct (with surface irregularly, weakly rugulose) to indistinct (surface rugose, disrupting carinate margin of areola); lateral propodeal areas posteriorly varying from nearly unsculptured and distinct to rugose and indistinct; propodeum largely bare medially, with a few scattered setae. Mesopleuron largely bare, with sparse setae in unsculptured subalar region and a small patch of setae dorsad mid coxa; posterior margin unsculptured. Precoxal sulcus weakly impressed but distinct; unsculptured. Metapleuron bare on dorsal half except for small patch below wing, with a few long setae medially, and patches of setae among rugulose sculpture along ventral margin and in groove on ventral half of anterior margin; otherwise unsculptured.

Wings. Fore wing stigma parallel-sided, discrete posteriorly, 7.50–7.75 × longer than wide; r1 arising from basal 0.35; 1RS (excluding parastigma) 0.20–0.25 × length of 1M; RS+M straight or nearly so; m-cu postfurcal, extending into basal corner of second submarginal cell; second submarginal cell weakly converging distally; 3RSa 1.10–1.25 × longer than 2RS; 2RS 2.5–3.4 × longer than r, the two not forming a continuous line; 2RS with distinct median bend; 3RSb very weakly bowed, nearly straight; 3M variable, but often pigmented and sclerotized for most of its length; 2CUa 0.5–0.7 × length of 2cu-a, 2CUb arising well above middle of first subdiscal cell; 1cu-a distad 1M by about 1.0 × its length; 1–1A bowed toward wing margin, and separated therefrom by its width. Hind wing RS a weak but distinct, unpigmented crease, extending nearly to wing margin in most specimens; 2M extending to wing margin as a more deeply impressed line, very weakly pigmented for much of its length; m-cu usually a deeply impressed, curved line extending about half distance to wing margin.

Metasoma distinctly petiolate; head 3.5–3.8 × wider than apex of T1. T1 2.15–2.35 × longer than apical width; nearly parallel-sided, with apex 1.20–1.35 × wider than base; surface striate throughout, above and below lateral carina; one or two very shallow, subapical depressions usually present dorsally; dorsope distinct, deep; laterope completely absent; dorsal carina present only at base, lateral carina usually distinct throughout; spiracle positioned 0.6 × length of T1 from the base; S1 extending about 0.25–0.30 × length of T1; dorsal surface of petiole in profile evenly convex from base to apex. T2 and following without sharp lateral margins; spiracle of second metasomal tergum laterally displaced, not visible in dorsal view. Ovipositor as long as mesosoma; ovipositor sheath 0.6–0.7 × length of mesosoma, with 2–3 irregular rows of long setae along its length.

Color: head, including antenna, mesosoma, petiole and ovipositor sheath dark brown except scape yellow; mandible, lower gena, ventral portion of clypeus, pedicel (occasionally), face adjacent antennal base, propleuron, anterior margin of pronotum, spot on mesopleuron below wing and a smaller spot above mid coxa, two streaks on either side of midpit on mesoscutum, posterior margins of scutellum and metapleuron, and petiole laterally (occasionally) dark yellow to orange; palps pale yellow, nearly white. Legs and metasoma beyond T1 yellow except hind tibia, hind tarsi, lateral margin of metasomal terga 2 + 3 and often anterior half of terga 4–6 brown, the hind tibia often paler medially.

*Male*. As in female except antenna with 41–45 flagellomeres, head 4.0–4.6 × wider than apex of T1 and T1 2.5–2.9 × longer than apical width. Body somewhat darker in color, with metasomal terga 6, 7, and most or all of 5 dark brown.

Body length 3.2–4.3 mm; wing length 3.5–4.2 mm.

#### Diagnosis.

**Figures 42–45.**
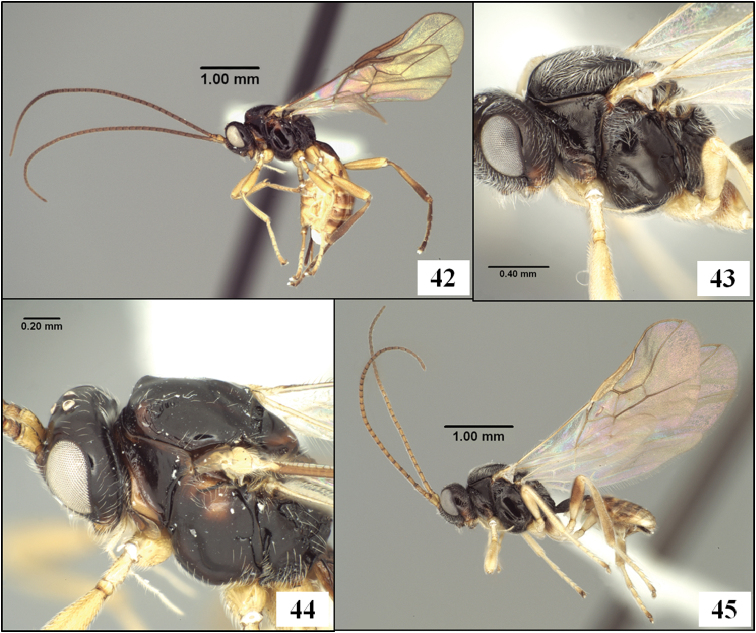
*Eurytenes* (*Stigmatopoea*) spp. **42** *ES*.) *maya* Wharton sp. n., paratype female, habitus **43** *ES*.) *norrbomi* Wharton sp. n., holotype female, mesosoma **44** *ES*.) *maya*, paratype female, head and mesosoma, dorsal-lateral view **45** *ES*.) *norrbomi*, holotype female, habitus.

This species runs to *Opius* (*Nosopoea*) in Fischer (1972, 1977) on the basis of the exposed labrum, distinct midpit on the mesoscutum, and absence of sculpture within the precoxal sulcus. It differs from described species placed in the subgenus *Nosopoea* by the combination of larger size, more numerous flagellomeres, relatively well-developed notauli (Fig. 44), parallel-sided T1 (Fig. 56), and parallel-sided stigma (Fig. 64), all characters which it shares with the type species of *Stigmatopoea*, *Eurytenes (Stigmatopoea) macrocerus*. In *Eurytenes maya* the anterior declivity of the mesoscutum is more vertical and the anterior-lateral corners of the mesoscutal disc are distinctly elevated (Fig. 44) in comparison to *Eurytenes macrocerus*. *Eurytenes maya* differs from the other species described below, *Eurytenes norrbomi*, sp. n., by the possession of a relatively longer ovipositor (Fig. 42 vs. Fig. 45) and a less densely setose mesoscutum (Fig. 44 vs. 43).

#### Biology.

All specimens were reared from Mexican populations of *Rhagoletis pomonella* (Walsh) infesting either hawthorns (species of *Crataegus* L.) or apples (*Malus domestica* Borkh.).

#### Etymology.

The species name is in reference the Mayan Indians of this region.

#### Remarks.

This species is similar in general appearance to members of the genus *Lorenzopius*, but T1 is not distinctly tubular as it is in the latter genus (see discussion below under *Lorenzopius*). The overall resemblance to *Lorenzopius* is enhanced by the presence of weak depressions on T1 that are similar in position in *Eurytenes maya* and *Lorenzopius calycomyzae* van Achterberg and Salvo (Figs 55, 56). The depressions are variable within members of the same reared series of *Eurytenes maya*: being absent, for example, in the holotype, but well developed in some of the paratypes.

The limited information on hosts suggests that species with a more tubular petiole, such as those in *Lorenzopius*, are parasitoids of leaf-mining Agromyzidae while the species of *Stigmatopoea* attack both leaf-mining and fruit-infesting tephritids.

**Figures 46–49.**
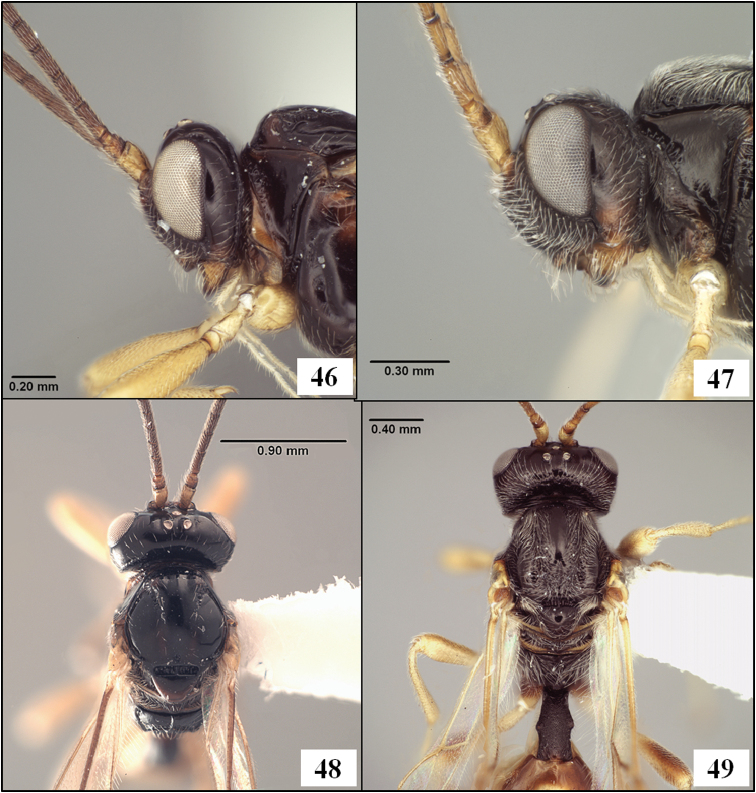
*Eurytenes* (*Stigmatopoea*) spp. **46** *ES*.) *maya* Wharton sp. n., paratype female, head, lateral view **47** *ES*.) *norrbomi* Wharton sp. n., holotype female, head, lateral view **48** *ES*.) *maya*, paratype female, head and mesosoma, dorsal view **49** *ES*.) *norrbomi*, holotype female, dorsal view.

### Eurytenes (Stigmatopoea) norrbomi

Wharton sp. n.

urn:lsid:zoobank.org:act:FC57AAB3-6290-4076-BB44-6289F8ABF2EC

http://species-id.net/wiki/Eurytenes_norrbomi

Figs 43, 45 47, 49 51, 53 57

#### Type locality.

Mexico, Morelos, Km. 9–10 between Huitzilac and Lago Zempoala.

#### Type material.

Holotype. Female (UNAM), first label, first line: MEXICO: Morelos second line: Km 9–10, btw. Huitzilac third line: & Lago Zempoala fourth line: roadside, 22–24.ix.1991 fifth line: A. L. Norrbom #42

**Paratypes:** Mexico, 4 females, same data as holotype (TAMU, USNM); 1 female, Mexico, Rt. 890, Km 9 area, 6 km W Lago Zempoala 2.x.1991, Norrbom, #43, reared ex. *Trypeta concolor* ex. leafmines on *Barkleyanthus salicifolius* (91M1D) (TAMU). 3 males, Distrito Federal, Rt. 95 (libre), Km 42–43, 1 km N. La Cima, 20–26.ix.1991 A. L. Norrbom, #41, reared ex. *Trypeta concolor* ex. leafmines on *Barkleyanthus salicifolius* (91M1) (TAMU, USNM).

#### Description.

*Female*. Head in dorsal view 1.2–1.3 × broader than mesoscutum, 1.75–1.85 × broader than face; eye in dorsal view 1.2–1.5 × longer than temple, temples weakly receding behind eyes. Frons and vertex as in *Eurytenes maya* except vertex and outer part of occiput densely covered with long, decumbent setae; width of ocellar field 1.20–1.35 × distance from ocellar field to eye. Face 1.75–1.85 × wider than high; slightly less polished than frons; uniformly setose (as in Figs 51, 53), distinctly punctate, the punctures separated by about 1 × their diameter. Frons and face delimited by a slightly more distinct change in sculpture in area between torulus and eye. Malar space about 0.6 × basal width of mandible, 0.25 × eye height. Clypeus 3.0–3.4 × wider than high; protruding in profile. Occipital carina distinctly curved medially at dorsal end, absent mid-dorsally, the space where the carina is absent approximating width of ocellar field. Antenna 1.15–1.30 ×longer than fore wing, with 31–33 flagellomeres; first flagellomere 1.05–1.10 × longer than second, 1.05–1.20 × longer than third; flagellomeres 3.1–4.1 × longer than wide basally, 2.3–2.7 longer than wide apically. Head otherwise as described for *Eurytenes maya*.

Mesosoma 1.35–1.45 × longer than high; 1.8–1.9 × longer than wide; 1.3–1.4 × higher than wide. Pronotum dorsally as in *Eurytenes maya* but with slightly enlarged pit in middle of crenulate groove consistently present; crenulae extending in shallow groove onto pronotum laterally, covering dorsal 0.2–0.6; groove margined anteriorly as in *Eurytenes maya*. Anterior declivity of mesoscutum completely vertical, densely covered with white, decumbent setae except for bare median band extending posteriorly to midpit; anterior-lateral corners of mesoscutum at upper edge of declivity elevated, rounded, densely setose, the setal pattern extending in broad bands all along notauli and laterally from anterior declivity to tegula; notauli complete, extending from anterior margin to scuto-scutellar sulcus, weakly converging posteriorly alongside but not into tear-drop shaped midpit; crenulate throughout, with sculpture extending laterally around margin to tegula, sculpture largely obscured by dense setae; lateral lobes of mesoscutum bare posterior-medially. Scuto-scutellar sulcus 4–5 × wider than midlength, lateral margins difficult to discern due to setal density; with low midridge and indistinct crenulae on either side; otherwise as in *Eurytenes maya*. Scutellum as in *Eurytenes maya* except with long marginal setae extending medially to cover most of posterior 0.5. Propodeum extensively rugulose, obscuring nearly all traces of carinae; pleural carina weak, often indistinct, very short median carina often present basally; transverse carina rarely weakly indicated across middle; propodeum uniformly setose anteriorly, with a few scattered setae posteriorly. Mesopleuron as in *Eurytenes maya* except subalar region densely setose and groove below subalar ridge varying from nearly smooth to weakly rugulose. Precoxal sulcus distinctly impressed, unsculptured. Metapleuron a little more extensively setose but otherwise as in *Eurytenes maya*.

Wings. Fore wing stigma parallel-sided, discrete posteriorly, 6.3–6.6 × longer than wide; r1 arising from basal 0.35; 1RS (excluding parastigma) 0.25–0.35 × length of 1M; RS+M weakly sinuate; 3RSa 1.05–1.30 × longer than 2RS; 2RS 2.6–3.1 × longer than r; 2RS and 3RSb straight; 3M variable, but often pigmented and sclerotized for most of its length; 2CUa 0.8–0.9 × length of 2cu-a, 2CUb arising slightly above middle of first subdiscal cell; position of m-cu, 1cu-a, and 1–1A, shape of second submarginal cell, and angle between r1 and 2RS as in *Eurytenes maya*. Hind wing as in *Eurytenes maya*.

Metasoma distinctly petiolate; head 3.75–4.10 × wider than apex of T1. T1 2.2–2.5 × longer than apical width; nearly parallel-sided, with apex 1.20–1.35 × wider than base; surface granular coriaceous throughout; completely without subapical depressions dorsally; dorsope, laterope, dorsal carinae, dorsal surface of T1 in profile, as in *Eurytenes maya*; lateral carina at least partially present but difficult to distinguish from surrounding sculpture. S1 extending about 0.25–0.30 × length of T1; T2 and following without sharp lateral margins; spiracle of second metasomal terga laterally displaced, only partially visible in dorsal view. Ovipositor shorter than mesosoma, base not visible in type series, but total length approximately 0.6–0.7 × length of mesosoma; ovipositor sheath 0.30–0.35 × length of mesosoma, with setal pattern as in *Eurytenes maya*.

Color: Mesosoma, T1, S1, ovipositor sheath, and most of head dark brown to black; antenna yellow basally, apical 0.3 brown; mandibles yellow; palps white; lower gena adjacent malar sulcus brown to brownish red; ventral 0.3–0.4 of clypeus yellow to brownish red. Tegula reddish brown with yellow margin. Legs yellow to pale yellow except most of hind coxa, apical 0.6–0.7 of hind femur, and fifth tarsomere of all legs brown; hind tibia varying from weakly infumate to light brown, basal 0.2 nearly always pale yellow. T2 mostly brownish red with median yellow blotch posteriorly; T3 yellow with anterior and lateral margins brownish red; T4-T6 yellow with anterior and lateral margins dark brown; visible parts of remaining terga yellow.

*Male*. As in female except antenna with 37 flagellomeres; eye in dorsal view 1.55–1.75 × longer than temple; width of ocellar field 1.05–1.10 × distance from ocellar field to eye. Color same except visible parts of apical terga dark brown.

Body length 2.8–3.5 mm; wing length 3.2–3.6 mm.

#### Diagnosis.

This species shares with *Eurytenes maya* and *Eurytenes macrocerus* the diagnostic features noted above for *Stigmatopoea*. *Eurytenes norrbomi* is most readily differentiated from *Eurytenes maya* on the basis of the more densely setose head and body (Figs 43, 47, 49), particularly the vertex, occiput, and mesoscutum, and the more extensively rugose propodeum. It also has a shorter ovipositor than *Eurytenes maya* (Fig. 45 vs. Fig. 42). The setal pattern on the mesoscutum also differentiates *Eurytenes norrbomi* from *Eurytenes macrocerus*. The latter has shorter setae that are more sparsely distributed laterally (Fig. 54).

#### Biology.

Four of the specimens from the type series were reared from puparia of *Trypeta concolor* (Wulp) (Tephritidae) mining leaves of *Barkleyanthus salicifolius* (H.B.K.) H. Robins & Brett (Asteraceae). The remaining specimens were collected from flowers of this same plant together with *Trypeta concolor* and *Trypeta reducta* Han and Norrbom. See Han and Norrbom (2005) for details on the hosts and the collecting localities.

#### Etymology.

This species is named after the collector, Allen Norrbom, who has provided many valuable host records for tephritid parasitoids.

#### Remarks.

This species attacks leaf-mining tephritids, as does *Eurytenes macrocerus*, while *Eurytenes maya* attacks fruit-infesting tephritids. Despite the difference in host habitat, all three species share many morphological features, and readily fit the characterization of *Eurytenes* (*Stigmatopoea*) as given above.

**Figures 50–53.**
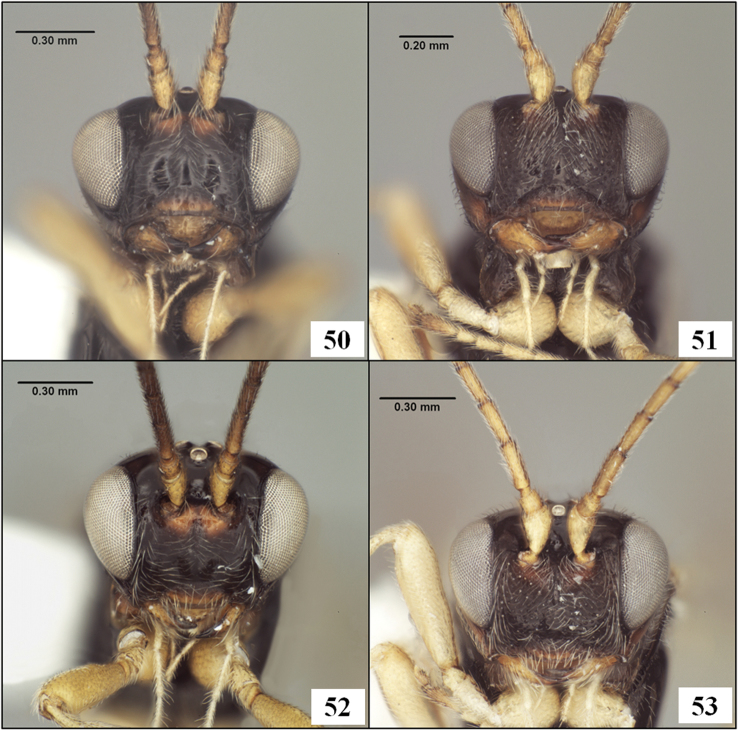
*Eurytenes* (*Stigmatopoea*) spp. **50** *ES*.) *maya* Wharton sp. n., paratype female, face, frontal view **51** *ES*.) *norrbomi* Wharton sp. n., holotype female, face, frontal view **52** *ES*.) *maya*, paratype female, face, slightly deflected **53** *ES*.) *norrbomi*, holotype female, face, slightly deflected.

**Figures 54–57.**
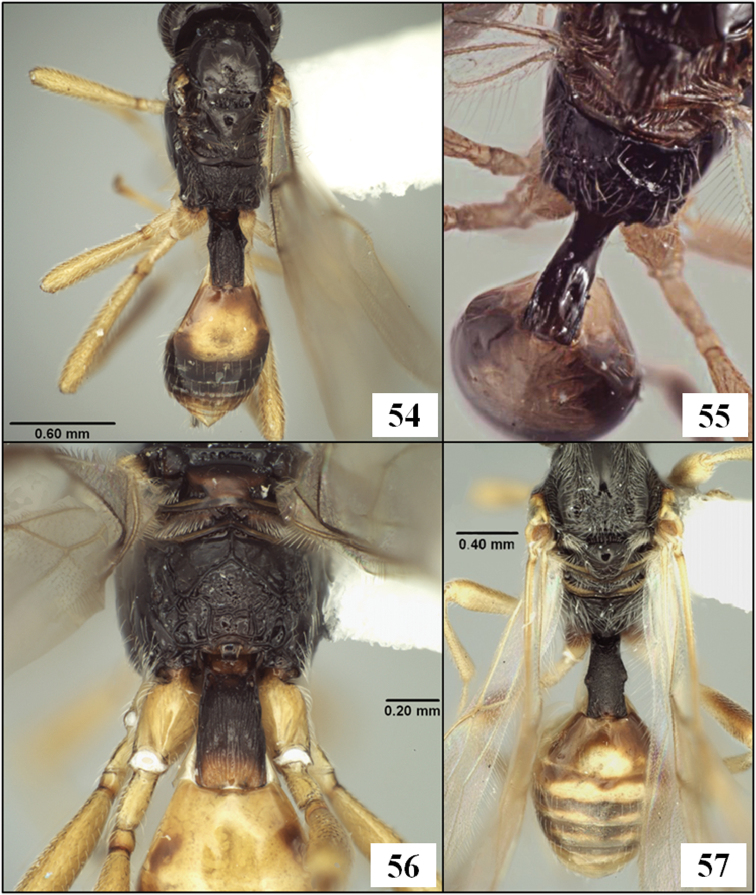
*Eurytenes* (*Stigmatopoea*) and *Lorenzopius*. **54** *ES*.) *macrocerus* (Thomson), mesosoma and metasoma, dorsal view **55** *Lorenzopius calycomyzae* van Achterberg and Salvo, holotype female, T1, dorsal view **56** *ES*.) *maya* Wharton sp. n., paratype female, propodeum and T1, dorsal view **57** *ES*.) *norrbomi* Wharton sp. n., holotype female, mesosoma and metasoma, dorsal view.

**Figures 58–61.**
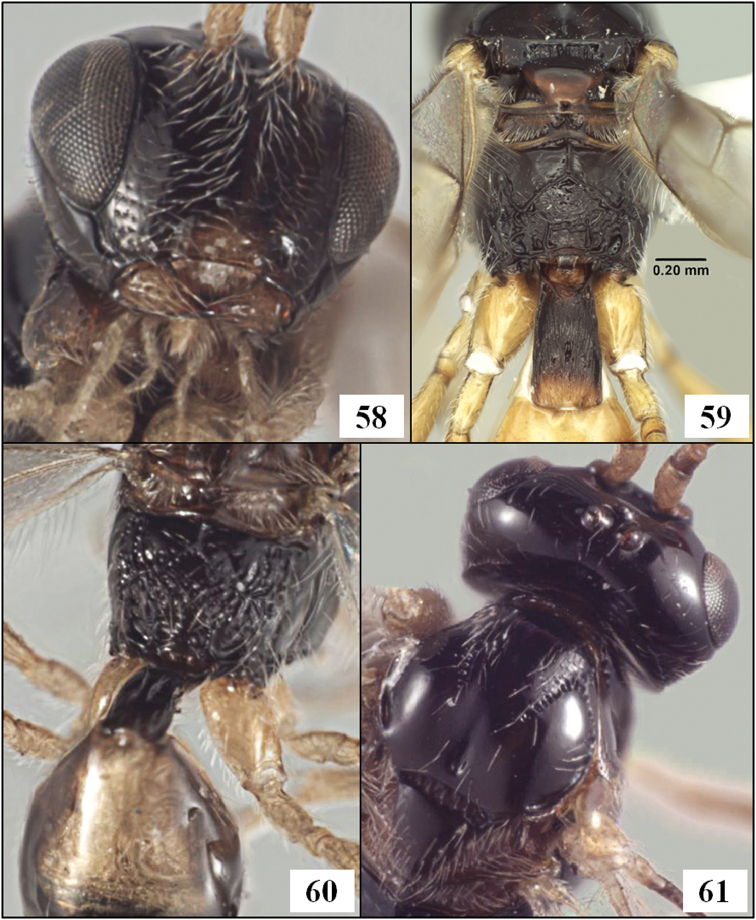
*Lorenzopius* and *Eurytenes* (*Stigmatopoea*). **58** *Lorenzopius calycomyzae* van Achterberg and Salvo, holotype female, face **59** *ES*.) *maya* Wharton sp. n., propodeum and T1 **60** *Lorenzopius calycomyzae*, propodeum **61** *Lorenzopius calycomyzae*, head and mesoscutum, dorsal view.

**Figures 62–65.**
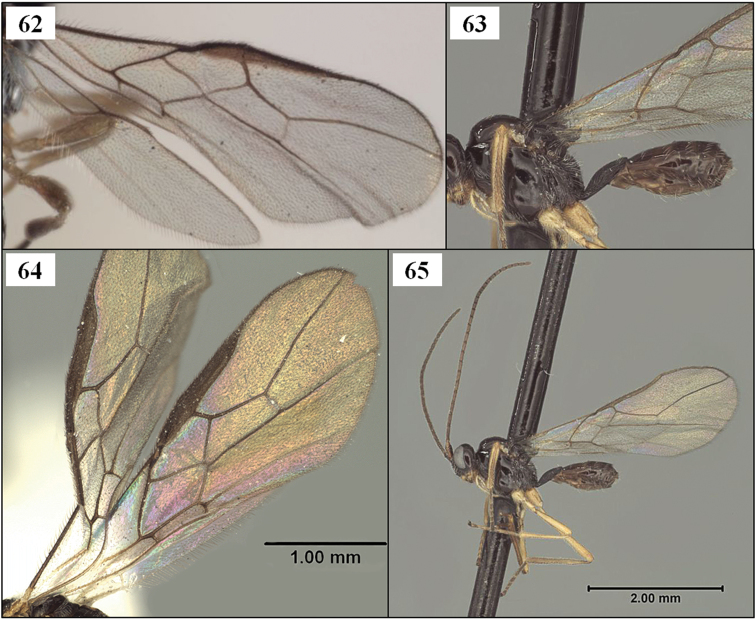
Opiinae. **62** *Lorenzopius calycomyzae* van Achterberg and Salvo, holotype female, wings **63 ***Tubiformopius tubigaster* (Fischer), holotype male, lateral view showing T1, S1, and wing base **64** *Eurytenes (Stigmatopoea) maya* Wharton, sp. n., paratype male, fore wing **65** *Tubiformopius tubigaster*, holotype male, habitus.

### Lorenzopius

van Achterberg & Salvo

http://species-id.net/wiki/Lorenzopius

Lorenzopius van Achterberg & Salvo, 1997: 190–192. Type species: *Lorenzopius calycomyzae* van Achterberg & Salvo, 1997. Original designation.

#### Diagnosis.

Mandible distinctly narrowed from base to apex, without basal lobe ventrally. Labrum exposed. Clypeus relatively flat, not distinctly protruding in profile; ventral margin sharp, truncate to weakly concave. Malar sulcus a sharp, weakly curved groove. Occipital carina broadly absent dorsally, present laterally; widely separated from hypostomal carina ventrally. First flagellomere longer than second. Propleuron ventral-laterally without oblique carina; pronotum dorsally without pronope or otherwise enlarged pit, posterior margin transversely rugulose. Notauli deep, narrow, well developed anteriorly, usually extending onto disc posteriorly; midpit present. Precoxal sulcus distinctly impressed. Propodeum with large areola, posterior portion often obscured by rugose sculpture. Fore wing stigma long, narrow, parallel-sided, discrete posteriorly, r1 arising distinctly basad its midpoint but not from extreme base; m-cu entering base of second submarginal cell; second submarginal cell with 2RS shorter than 3RSb; 2CUb arising above middle of hind margin of first subdiscal cell. Dorsope and laterope of T1 absent; S1 at least 0.7 × length of T1 in females, slightly shorter in males, apparently fused to T1; T1 long and narrow throughout; T2 and following terga unsculptured. Ovipositor tapering evenly to a fine point, without dorsal nodes or ridges.

#### Remarks.

*Lorenzopius* and *Tubiformopius* are both characterized by having a tubular petiole with a long S1 which appears fused to T1 (Figs 6, 8). In the material available, S1 is longer in *Lorenzopius* than in *Tubiformopius* but there are more significant differences in the shape of the mandible, wing venation, and mesoscutal sculpture, as noted above in the section discussing genus group characters. *Lorenzopius* also shares many features with *Eurytenes* (*Stigmatopoea*), but the petiole is less tubular in the latter, with a distinctly shorter S1 that is clearly separated by membrane from T1 (Fig. 5).

The shape of the stigma has been proposed as a useful feature for assessing relationships among opiines (Wharton 1988), and both *Lorenzopius* and *Stigmatopoea* have the stigma more or less parallel-sided or slightly expanded distally. Unfortunately, the stigma often curls as specimens dry after death, and this feature then not only becomes difficult to assess properly, but is often illustrated in the curled position giving a misleading impression of the true form. For example, the shape of the stigma is difficult to discern on the holotype of *Lorenzopius calycomyzae* (Fig. 62). However, the shape is more readily discernible in the holotype of *Lorenzopius tubulatus* (Fig. 68) and in several other specimens of *Lorenzopius* available for examination (from CNC and TAMU), and these clearly show a parallel-sided stigma.

**Figures 66–69.**
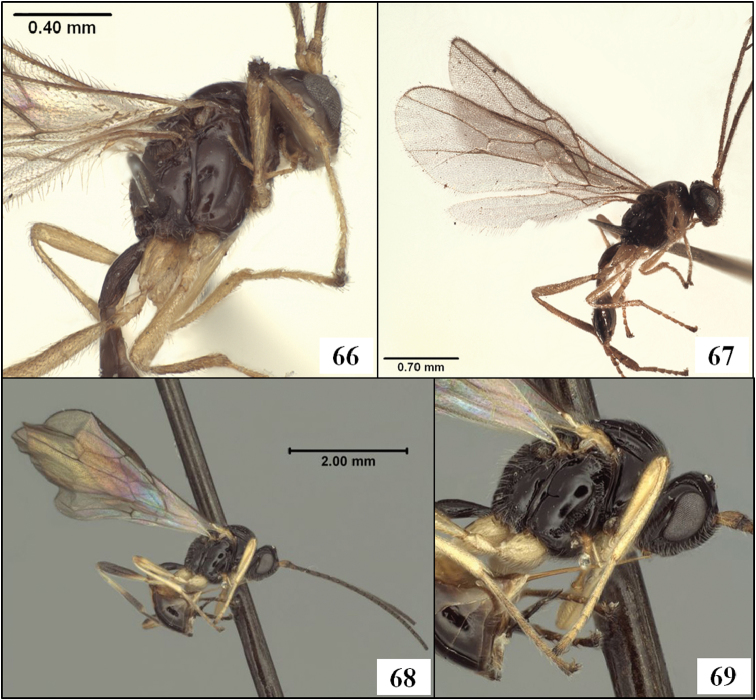
*Lorenzopius* spp. **66** *Lorenzopius euryteniformis* (Fischer), holotype male, dorsal-lateral view showing unsculptured precoxal sulcus **67** *Lorenzopius euryteniformis*, holotype male, habitus **68** *Lorenzopius tubulatus* (Fischer), holotype female, habitus **69** *Lorenzopius tubulatus*, holotype female, mesosoma, lateral view showing sculptured precoxal sulcus.

We recognize two distinct species groups within *Lorenzopius*: the *calycomyzae* species group containing the orginially included species *Lorenzopius calycomyzae*, *Lorenzopius tubulatus*, and *Lorenzopius sanlorenzensis* and a second group typified by *Lorenzopius euryteniformis* (Fischer), new combination. All have same basic wing venation and petiole. The precoxal sulcus is distinctly sculptured in the *calycomyzae* species group (Fig. 69) but the distinctly impressed sulcus is unsculptured or nearly so in the *euryteniformis* species group (Fig. 66). The smallest specimens of the *calycomyzae* species group examined during this study are slightly larger than the largest available specimens of the *euryteniformis* species group and perhaps as a consequence they tend to have slightly longer notauli and more sculpture bordering the supra-marginal carina extending from the base of the notaulus to the tegula. Most of the species we have examined from the *euryteniformis* species group have reduced propodeal sculpture with the areola clearly visible (Figs 72, 73). In addition to holotypes of *Lorenzopius tubulatus* and *Lorenzopius sanlorenzensis* and the holotype and paratypes of *Lorenzopius calycomyzae*, we have seen two additional specimens from Argentina (TAMU), and one specimen each from Peru and Costa Rica (both CNC) representing the *calycomyzae* species group. RAW has examined 17 specimens representing the *euryteniformis* species group in addition to the holotype of *Lorenzopius euryteniformis*. The material examined includes specimens housed in TAMU and CNC collected in Bolivia, Colombia, Costa Rica, Dominican Republic, Guatemala, and Mexico (as far north as Monterrey in Nuevo Leon).

**Figures 70–73.**
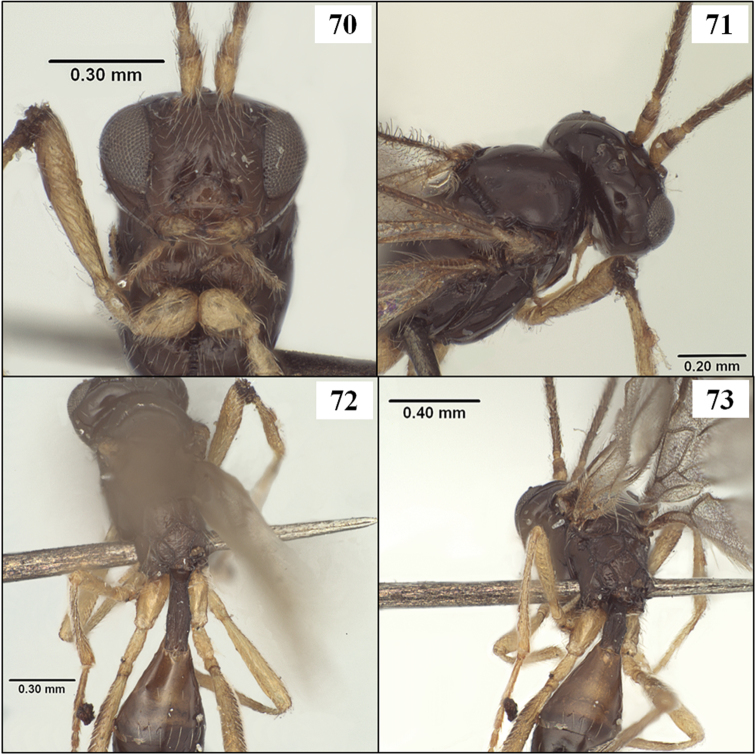
*Lorenzopius euryteniformis*, holotype male. **70** face **71** mesoscutum and head, dorsal-lateral view **72** propodeum and T1–3 **73** propodeum and metasoma.

Lengthy descriptions (Fischer 1963, 1964, 1979, van Achterberg and Salvo 1997) and some redescriptions (Fischer 1977) are available for the described species of *Lorenzopius* and van Achterberg and Salvo (1997) provide a useful key to the species of the *calycomyzae* species group. Species pages for *Lorenzopius calycomyzae* (Figs 6, 55, 58, 60–62), *Lorenzopius tubulatus* (Figs 3, 68, 69) and *Lorenzopius euryteniformis* (Figs 66, 67, 70–73) can be found at http://peet.tamu.edu/projects/8/public/site/wharton_lab/home. The described species are readily differentiated. T1 is exceptionally long and narrow in *Lorenzopius tubulatus* (at least 4 × longer than apical width) and this species has darker legs than the others, with most of the hind femur dark brown. T1 is about 3 × longer than apical width in the other two species of the *calycomyzae* species group and the hind femora are yellow. The presence of a pair of pits on T1 is thus far a unique feature of *Lorenzopius calycomyzae* within *Lorenzopius* and this species is also characterized by orange markings dorsally in the middle of the mesosoma. The metasoma is darker in *Lorenzopius sanlorenzensis*, with T2+3 dark brown in this species and largely yellow in the other two members of the *calycomyzae* species group. *Lorenzopius euryteniformis* lacks sculpture within the depression of the precoxal sulcus.

The type species of *Lorenzopius* was described from specimens reared from *Calycomyza mikaniae* Spencer, a leafminer in the family Agromyzidae. RAW has also seen specimens from Colombia of a species nearly identical to *Lorenzopius euryteniformis* that was also reared from an agromyzid leafminer. No other host records are known for this genus but given the general similarity of the habitus and the length and shape of the ovipositor, we predict that other species will also prove to be agromyzid leafminer parasitoids.

### Lorenzopius euryteniformis

Fischer, 1963 comb. n.

http://species-id.net/wiki/Lorenzopius_euryteniformis

Figs 66, 67 70–73

Opius euryteniformis Fischer, 1963: 288–290. Holotype male NHMW (examined).
Opius (Nosopoea) euryteniformis : Fischer 1977: 195, 206–208.

#### Type locality.

Costa Rica, Mount Irazu, 2200–2300 m.

#### Type material.

Holotype. Male (NHMW), first label, first line: Costa Rica, Irazu, second line: 2200–2300 m, 21–28. third line: V.’30. Reimoser Second label, first line: Opius second line: euryteniformis third line: sp. n. fourth line: det. Fischer Third label: Holotype [purple], Fourth label: NHMW

#### Diagnosis.

Holotype male. Head in dorsal view with temples neither receding nor expanded beyond eyes; in lateral view, eye about 1.6 × longer than temple. Labrum partly exposed between clypeus and mandibles (Fig. 70); clypeus about twice as wide as tall, flat or nearly so, not distinctly protruding in profile, ventral margin truncate to very weakly concave. Mandible without basal lobe. Malar space well developed, longer than basal width of mandible; malar sulcus deeply impressed. Antenna with 27 flagellomeres. Pronotum dorsally not visible in holotype. Disc of mesoscutum nearly bare, with scattered setae along margin of anterior declivity and a single pair of setae arising about midlength of notauli; notaulus extending posteriorly along anterior 0.3 of disc, less than half distance to small, deep, round midpit; supra-marginal carina distinct anteriorly, not extending to level of tegula. Scuto-scutellar sulcus relatively narrow (Fig. 71), densely crenulate throughout. Precoxal sulcus distinctly impressed, long, narrow, completely unsculptured. Propodeum largely smooth with broad, pentagonal areola on posterior 0.65, anterior 0.35 with median carina. Fore wing stigma long, narrow, with some postmortem curling, but at least 4.5 × longer than width at r1; r1 arising from basal 0.3; second submarginal cell long, weakly converging distally, 3RSa 1.7 × longer than 2RS; 1RS 0.2 × length of 1M; m-cu postfurcal; 2CUb arising a little above middle of hind margin of first subdiscal cell, 2cu-a present, tubular. T1 long, narrow, apparently fused ventrally with S1 for most of its length, 4x longer than apical width, apex as wide as base; surface completely striate. T2 and following smooth, polished.

#### Biology. 

Unknown.

#### Remarks.

Placement of this species in *Lorenzopius* is based on the wing venation and long S1, which is 0.65 × length of T1 in the male holotype; S1 appears fused to T1. See additional comments on species groups under the remarks section for the genus.

The holotype bears a single data label containing the information given above. However, the label data listed in the original description are as follows: “Costa Rica, La Caja bei San José, H. Schmidt”. As this species was described from a single male specimen, and the specimen from Irazu labeled as the holotype matches the original description, it is likely that the locality data in the original publication is an inadvertent error. The new species described immediately before *euryteniformis* in the same publication is from the La Caja locality. The type locality should therefore be Irazu (a mountain in Costa Rica), somewhere in the 2200–2300 m range in elevation.

### Opius

Wesmael

http://species-id.net/wiki/Opius

Opius Wesmael, 1835: 115. Type species: *Opius pallipes* Wesmael, 1835. Subsequent designation (Wharton 1987b, ICZN 1988).

#### Remarks.

Van Achterberg and Salvo (1997) restricted the name *Opius* to species with a basal lobe on the mandible, referring to a classification in press that has yet to be published. A major concern in this regard is that the type species of *Biosteres* Foerster, another large genus within the Opiinae, also has a basal mandibular lobe. Until a more complete classification is offered, we prefer to treat *Opius* in a much broader sense as a repository for the bulk of the Opiinae whose relationships remain uncertain, largely following the approach of Fischer (1972) and Wharton (1997).

Diagnoses are presented below for two species that represent a fairly diverse group of neotropical Opiinae that differ from both *Lorenzopius* and *Tubiformopius* in several features. These species all have a narrow, parallel-sided T1 and distinctly visible S1, though S1 is never as long as in *Lorenzopius*, and seldom as long as in *Tubiformopius*. Ultimately, the relationships of genus group taxa such as *Eurytenes* s.l., *Lorenzopius*, and *Tubiformopius* will have to be carefully considered in order to place the many neotropical species with a distinct S1.

### Opius incoligma

Fischer

http://species-id.net/wiki/Opius_incoligma

Figs 74–77 81

Opius (Nosopaeopius) incoligma Fischer, 1979: 274–276. Holotype female AEIC (examined).
Opius (Nosopaeopius) incoligma : Yu et al. 2005, 2012 (electronic catalogs).

#### Type locality.

Colombia, Magdalena, 41 km south of Sta. Marta, 7000 ft.

#### Type material.

Holotype. Female (AEIC), first label, first line: 41Km S.St. Marta second line: Magd., Colombia third line: V.6.1973 7000 ft. fourth line: Howden&Campbell second label [red]: Holotype third label, first line: [female symbol] Opius second line: incoligma third line: Holotype fourth line: det Fischer sp. n.

#### Diagnosis.

Holotype female. Labrum completely concealed by mandibles; clypeus nearly as tall as wide, flat, not protruding, ventral margin convex. Mandible without basal lobe, distinctly narrowing apically to narrow, bifid tooth. Malar space distinct, malar sulcus deep, distinct. Antenna with 33 flagellomeres. Pronotum dorsally without pronope or distinct pit, mostly unsculptured, crenulate posterior margin broadly interrupted medially. Disc of mesoscutum nearly bare, with a few setae along traces of notauli; midpit small, distinct, narrowly elongate; notauli weak, present as very short, weakly sculptured grooves directed posterior-medially from and along edge of anterior declivity, not extending posteriorly onto disc of mesoscutum; distinct supra-marginal carina extending laterally from base of notaulus to tegula. Scuto-scutellar sulcus narrow (about 6–7 × wider than long but difficult to measure), crenulate throughout. Precoxal sulcus distinct, moderately deep, long, completely unsculptured, somewhat vertically oriented as in *Lorenzopius*. Propodeum granular rugose, with very short median carina anteriorly, densely setose throughout. Fore wing stigma parallel-sided to weakly expanded apically; r1 longer than stigma width; second submarginal cell long, weakly narrowing distally; m-cu weakly postfurcal; 2CUb arising distinctly above middle of first subdiscal cell, 2CUa nearly absent. Hind coxa smooth; hind femur slender, weakly bilobed. T1 weakly strigose, irregularly sculptured with smooth patches; dorsal carina short but distinct; lateral carina very well developed, extending from junction with dorsal carina to apex, passing ventrad spiracle; dorsope shallow, indistinct, laterope shallow, weakly indicated by a long, narrow groove; T1 spiracle situated slightly posteriad midlength of T1; T1 narrow, parallel-sided, 2.6 × longer than apical width; no visible membrane between S1 and T1, though lateral margin between the two clearly visible; S1 0.35 × length of T1.

#### Remarks.

**Figures 74–77.**
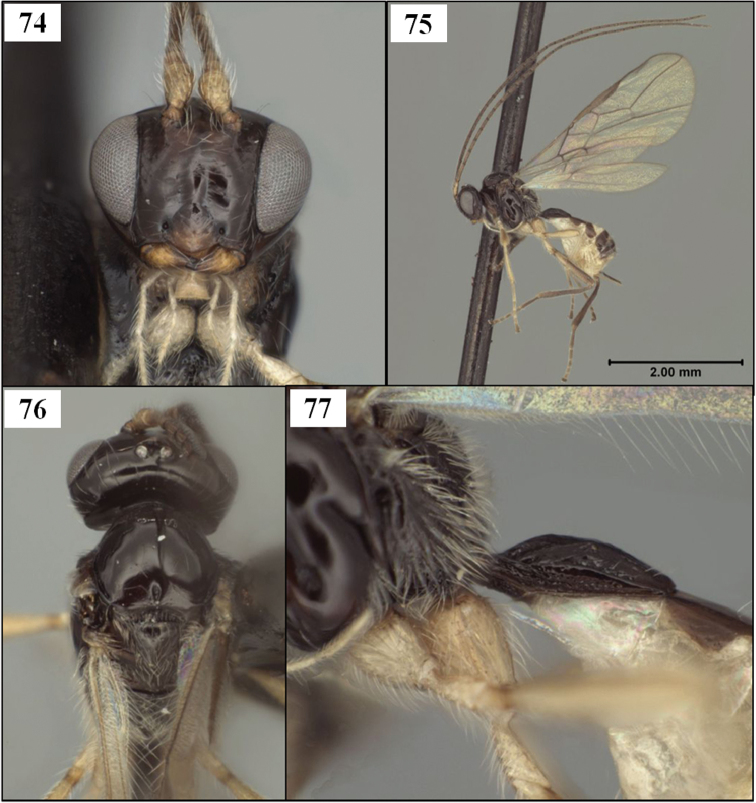
*Opius incoligma* Fischer, holotype female. **74** face **75** habitus **76** head and mesonotum, dorsal view **77** T1, lateral view.

The venation (Fig. 75) and features of the first metasomal segment (Figs 77, 81) suggest a relationship to *Eurytenes* (*Stigmatopoea*), but this species differs most remarkably by the completely concealed labrum (Fig. 74). Also, unlike the other species of *Eurytenes*, *Lorenzopius*, and *Tubiformopius* treated here, the individual flagellomeres are long throughout in *Opius incoligma* but notably decreasing in length in the other species.

**Figures 78–81.**
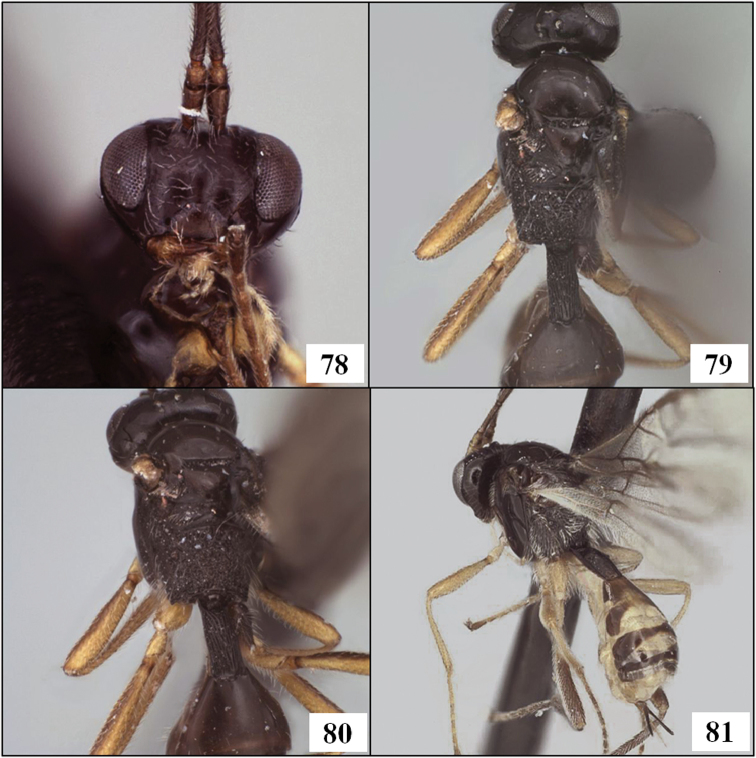
*Opius* spp. **78** *Opius rugicoxis* Fischer, holotype female, face **79** *Opius rugicoxis*, holotype female, dorsal view **80** *Opius rugicoxis*, holotype female, propodeum and T1 **81** *Opius incoligma* Fischer, holotype female, dorsal-lateral view.

### Opius rugicoxis

Fischer

http://species-id.net/wiki/Opius_rugicoxis

Figs 78–80 82, 83

Opius rugicoxis Fischer, 1969: 251–254. Holotype female in AEIC (examined).
Opius (Stomosema) rugicoxis : Fischer 1977: 223, 248–249 (key, redescription); Yu et al. 2005, 2012 (electronic catalogs).

#### Type locality.

Ecuador, Troya, 2900 m.

#### Type material.

Holotype. Female (AEIC), first label, first line: Troya, Ecuador second line: VI. 10–13. 65 2900m. third line: Luis Pena second label [purple]: Holotype third label, first line: Opius [female symbol] second line: rugicoxis third line: det Fischer sp. n. fourth label, first line: Type no. second line: 659

#### Diagnosis.

**Figures 82–83.**
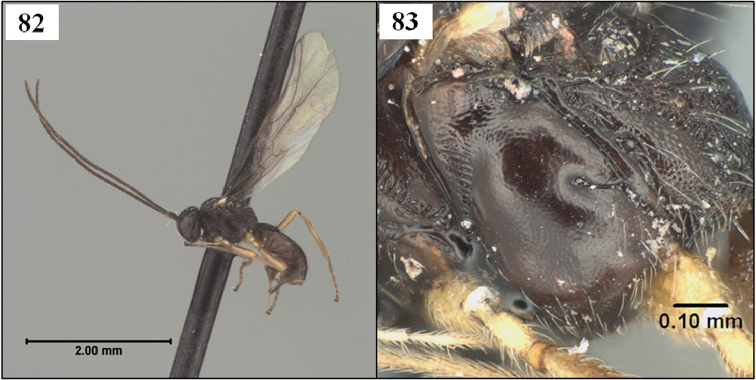
*Opius rugicoxis* Fischer, holotype female. **82** habitus **83** mesopleuron.

Holotype female. Labrum completely concealed by mandibles (Fig. 78); clypeus tall, flat, not protruding, ventral margin truncate. Mandible with broad, discrete basal lobe, apical half narrow, nearly parallel-sided. Malar space distinct; malar sulcus weak but present. Antenna with 25 flagellomeres. Pronotum not visible dorsally. Disc of mesoscutum (Figs 79, 80) bare, midpit small, round; notauli weak, present as very short, weakly sculptured grooves directed posterior-medially from and along edge of anterior declivity, not extending posteriorly onto disc of mesoscutum; weak supra-marginal carina extending laterally from base of notaulus nearly to tegula. Scuto-scutellar sulcus narrow (5–6 × wider than long), crenulate throughout. Precoxal sulcus absent, thus unsculptured (Fig. 83). Propodeum (Fig. 80) completely granular rugose, without carinae, very sparsely setose. Fore wing (Fig. 82) with stigma folded, shape not readily discernible; r1 shorter than stigma width; second submarginal cell long, distinctly narrowing distally; m-cu distinctly postfurcal; 2CUb arising below middle of first subdiscal cell. Hind coxa granular-rugose, hence the species name; hind femur slender, distinctly bilobed. T1 (Figs 79, 80) completely striate, the striae curving medially from basal-lateral area adjacent dorsal tendon attachment, obscuring dorsal and lateral carinae; dorsope absent, laterope not apparent; T1 spiracle indistinct, situated posteriad midlength of T1; T1 nearly parallel-sided, 2.25 × longer than apical width; S1 appears fused to T1; S1 0.3 × length of T1.

#### Remarks.

Fischer (1977) placed this species in his subgenus *Opius* (*Stomosema*), which he earlier (Fischer 1972) characterized on the basis of three features: a concealed labrum, absence of a mesoscutal midpit, and presence of sculpture in the precoxal sulcus. Unfortunately, the holotype has a small, shallow, but distinct midpit (Figs 79, 80) and lacks a precoxal sulcus (Fig. 83). This species would therefore key to *Opius* (*Nosopaeopius*) in Fischer (1972) and Fischer (1999). Regardless of subgeneric assignment, this species falls within *Opius* in the classifications of Fischer (1977, 1999), van Achterberg and Salvo (1997), and Wharton (1997). The shape and sculpture of the first metasomal segment and the relatively long S1 suggest a relationship to *Tubiformopius*, but I exlude this species from *Tubiformopius* for the present time primarily on the basis of wing venation and from *Lorenzopius* on the basis of the form of the mandible.

The hind coxa is smooth to weakly punctate in other species treated here.

### Tubiformopius

Fischer stat. rev.

http://species-id.net/wiki/Tubiformopius

Tubiformopius Fischer, 1998: 26. Type species: *Opius tubigaster* Fischer, 1968. Original designation.

#### Diagnosis.

Mandible very weakly narrowing, nearly parallel-sided over distal 0.5, more abruptly widening basally, with weak to distinct basal lobe. Labrum narrowly exposed to concealed. Clypeus relatively weakly but distinctly protruding in profile; ventral margin truncate. Malar sulcus absent or represented only by a short, weak indentation adjacent eye; malar space distinct, at least as long as basal width of mandible. Occipital carina broadly absent dorsally, present laterally, distinctly separate from hypostomal carina ventrally. First flagellomere much longer than second. Propleuron ventral-laterally without oblique carina. Notauli short, shallow, narrow, confined to anterior declivity, not extending onto disc posteriorly; distinct midpit absent. Precoxal sulcus broad, very weakly impressed, unsculptured. Propodeum granular rugose, without areola. Fore wing stigma long, narrow, curled in holotypes of both species treated below, but not as discrete distally as in *Lorenzopius* and *Stigmatopoea*; r1 arising distinctly basad midpoint of stigma but not from extreme base; m-cu entering first submarginal cell, widely separated from second submarginal cell; second submarginal cell with 2RS much shorter than 3RSb; 2CUb arising near middle of hind margin of first subdiscal cell, the posterior-distal corner of the latter broadly open. Dorsope and laterope of T1 absent; S1 about 0.5–0.6 × length of T1, apparently fused to T1; T1 long and narrow throughout; T2 and following terga unsculptured. Ovipositor not tapering evenly to a fine point.

**Remarks**. The diagnosis above is based on the holotypes of *Tubiformopius tubigaster* (Fischer) and *Tubiformopius tubibasis* (Fischer), new combination.

Fischer’s (1998) original description of *Tubiformopius* was very brief since it was only included in a key to the eight genera he treated in the *Opius* genus group. Although two species are indicated in the relevant couplet, only one, designated as the type species, is specifically named. As noted above under the general discussion of genus-group characters, Fischer (1999), without discussion, treated *Tubiformopius* as a synonym of *Lorenzopius*. Aside from the original descriptions and Fischer’s (1999) subsequent synonymy, neither *Tubiformopius* nor *Lorenzopius* has been further treated until now. We retain *Tubiformopius* as a valid genus distinct from *Lorenzopius* primarily on the basis of strong differences in the shape of the mandible (Fig. 85), fore wing venation (Figs 63, 65), and the notauli (Figs 86, 87). Fischer (1978) originally placed *Tubiformopius tubibasis* in *Opius* s.s. Differences in venation and the first metasomal segment (especially the long and apparently fused S1) separate *Tubiformopius* from *Opius* s.s. Fischer (1977) placed *Opius tubigaster* in the subgenus *Allophlebus* Fischer, 1972 but the type species of *Allophlebus* has T1 distinctly broadening apically with a very short, clearly separated S1, a distinct laterope, and the fore wing m-cu is postfurcal.

There is as yet no host data for either of the species currently included in *Tubiformopius*.

### Tubiformopius tubigaster

Fischer stat. rev.

http://species-id.net/wiki/Tubiformopius_tubigaster

Figs 8 63, 65 85–87

Opius tubigaster Fischer, 1968: 463–464, 483–485. Holotype male AEIC.
Opius (Allophlebus) tubigaster : Fischer 1977: 223, 248–249 (key, redescription).
Tubiformopius tubigaster : Fischer 1998: 26.
Lorenzopius tubigaster : Fischer 1999: 282; Yu et al. 2005, 2012 (electronic catalogs).

#### Type locality.

Ecuador, Cerro Tinajillas, 3200 m.

#### Type material.

Holotype. Male (AEIC), first label, first line: Cerro Tinajillas second line: 3200m Ecuador third line: III. 18–21. 65 fourth line: Luis Peña second label [purple]: Holotype third label, first line: Opius [male symbol] second line: tubigaster third line: det Fischer sp. n. fourth label: first line: Type no. second line: 589

#### Diagnosis.

Holotype male. Labrum partly concealed by mandibles (Fig. 85); clypeus nearly twice as wide as tall, protruding in profile, ventral margin truncate to very weakly concave. Mandible with basal lobe, apically nearly parallel-sided. Malar space distinct, malar sulcus not evident except as a small impression adjacent eye. Antenna with 26 flagellomeres. Pronotum dorsally not readily visible in holotype. Disc of mesoscutum nearly bare, with a sparse row of setae between notauli and transscutal articulation; midpit completely absent; notauli weak, present as very short, unsculptured grooves on anterior declivity, not extending posteriorly onto disc of mesoscutum; supra-marginal carina between base of notaulus and tegula absent. Scuto-scutellar sulcus relatively narrow (Figs 86, 87), crenulate throughout. Precoxal sulcus indistinct, short, broad, very shallow, completely unsculptured. Propodeum granular rugose, without median carina anteriorly, moderately setose. Fore wing stigma long, curled in holotype, but appears to be very gradually tapered distally; r1 equal to or slightly longer than stigma width; second submarginal cell long, distinctly narrowing distally; m-cu widely antefurcal (Fig. 63, 65); 2CUb arising about middle of hind margin of first subdiscal cell, 2cu-a absent, first subdiscal cell broadly open at posterior-distal corner. Hind coxa smooth; hind femur very long, slender, weakly bilobed. T1 (Figs 63, 65, 86, 87) completely striate, the striae curving medially from basal-lateral area adjacent dorsal tendon attachment, completely obscuring dorsal and lateral carinae; dorsope and laterope absent; T1 spiracle indistinct, situated posteriad midlength of T1; T1 nearly parallel-sided, 2.1 × longer than apical width; S1 appears fused to T1; S1 0.5 × length of T1.

**Remarks.** This species is very similar to *Tubiformopius tubibasis*, but differs in having a little more of the labrum exposed between the apex of the clypeus and the tightly closed mandibles. The hind coxae are yellow in *Tubiformopius tubigaster* and distinctly infumate in *Tubiformopius tubibasis*. Both species were described from Ecuador.

### Tubiformopius tubibasis

Fischer comb. n.

http://species-id.net/wiki/Tubiformopius_tubibasis

Fig. 84

Opius (Opius) tubibasis Fischer, 1978: 163–165. Holotype female in AEIC.
Opius (Opius) tubibasis : Yu et al. 2005, 2012 (electronic catalogs).

#### Type locality.

Ecuador, Cañar, Naupán, 3200 m.

#### Type material.

Holotype. Female (AEIC), first label, first line: W. Naupán(Cañar) second line: 3200m. Ecuador third line: XII. 10. 70 fourth line: Luis Peña second label [red]: Holotype third label, first line: [female symbol] Opius second line: tubibasis third line: Holotype sp. n. fourth line: det. Fischer fourth label [yellow] Type 1195

#### Diagnosis.

Holotype female. Labrum completely concealed by mandibles; clypeus tall, narrow, weakly protruding in profile, ventral margin truncate. Mandible with weak basal lobe, apically nearly parallel-sided. Malar space distinct, malar sulcus not evident except as a small impression adjacent eye. Antenna with 24 flagellomeres. Pronotum dorsally not readily visible in holotype. Disc of mesoscutum nearly bare, with a very sparse row of setae between notauli and transscutal articulation; midpit absent or nearly so, with faint indication of a depression when viewed in certain angles; notauli weak, present as short, weakly sculptured grooves on anterior declivity, not extending posteriorly onto disc of mesoscutum; supra-marginal carina between base of notaulus and tegula absent. Scuto-scutellar sulcus relatively narrow as in *Opius tubigaster*, crenulate throughout. Precoxal sulcus barely visible as a short, broad, very shallow, completely unsculptured indentation. Propodeum granular rugose, without median carina anteriorly, moderately setose. Fore wing with stigma long, curled in holotype, but very gradually tapered distally; r1 equal to or slightly longer than stigma width; second submarginal cell long, distinctly narrowing distally; m-cu widely antefurcal; 2CUb arising slightly below middle of hind margin of first subdiscal cell, 2cu-a absent, first subdiscal cell broadly open at posterior-distal corner. Hind coxa smooth; hind femur very long, slender, weakly bilobed. T1 completely striate, the striae curving medially from basal-lateral area adjacent dorsal tendon attachment, completely obscuring dorsal and lateral carinae; dorsope and laterope absent; T1 spiracle indistinct, situated at 0.65 length of T1; T1 parallel-sided, 2.5 × longer than apical width; S1 appears fused to T1; S1 0.6 × length of T1.

#### Remarks.

Van Achterberg and Salvo (1997) suggested the possibility that *tubibasis* might belong in *Lorenzopius* despite the absence of a midpit on the mesoscutum. The subsequently described *Tubiformopius* is a better fit because *tubibasis* is nearly identical to the type species of *Tubiformopius*, especially with respect to critical features of mesosomal sculpture and fore wing venation in addition to the shape of the mandible.

**Figures 84–87.**
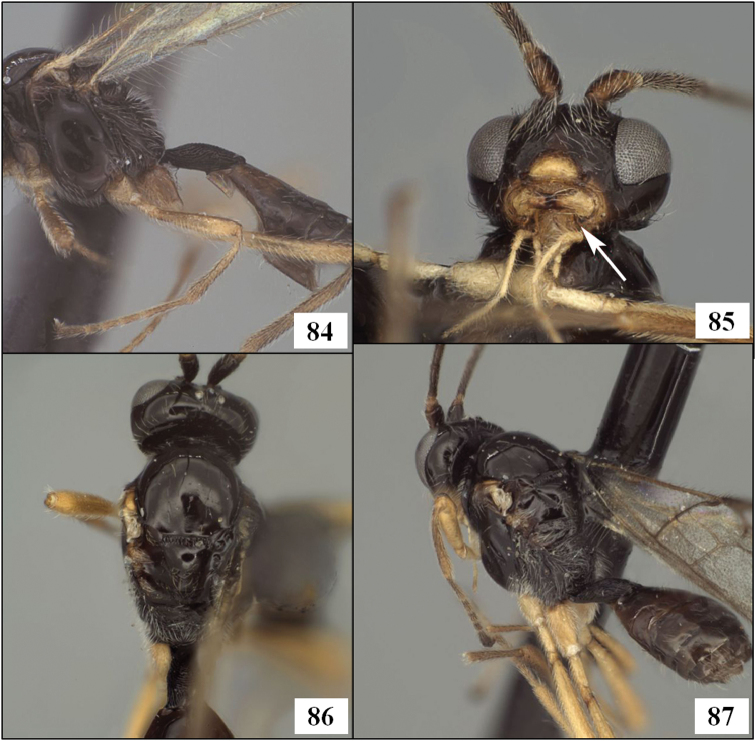
Tubiformopius spp. **84** T. tubibasis (Fischer), holotype female, T1, lateral view **85** T. tubigaster (Fischer), holotype male, face, arrow showing basal lobe of mandible **86** T. tubigaster, holotype male, head and mesoscutum, dorsal view **87** T. tubigaster, holotype male, dorsal-posterior view.

## Supplementary Material

XML Treatment for Diachasmimorpha

XML Treatment for Diachasmimorpha hildagensis

XML Treatment for Diachasmimorpha martinalujai

XML Treatment for Diachasmimorpha norrbomi

XML Treatment for Diachasmimorpha mexicana

XML Treatment for Diachasmimorpha sanguinea

XML Treatment for Eurytenes

XML Treatment for Eurytenes (Stigmatopoea) maya

XML Treatment for Eurytenes (Stigmatopoea) norrbomi

XML Treatment for Lorenzopius

XML Treatment for Lorenzopius euryteniformis

XML Treatment for Opius

XML Treatment for Opius incoligma

XML Treatment for Opius rugicoxis

XML Treatment for Tubiformopius

XML Treatment for Tubiformopius tubigaster

XML Treatment for Tubiformopius tubibasis

## Appendix

Hymenoptera Anatomy Ontology Table. (doi: 10.3897/zookeys.243.3990.app) File format: Microsoft Exsel file (xls). **Explanation note:** Morphological terms used in text, referenced to the Hymenoptera Anatomy Ontology (HAO).
